# How urban densification shapes walking behaviours in older community dwellers: a cross-sectional analysis of potential pathways of influence

**DOI:** 10.1186/s12942-020-00210-8

**Published:** 2020-04-16

**Authors:** Ester Cerin, Anthony Barnett, Casper J. P. Zhang, Poh-chin Lai, Cindy H. P. Sit, Ruby S. Y. Lee

**Affiliations:** 1 Mary MacKillop Institute for Health Research, Australian Catholic University, Level 5, 215 Spring Street, Melbourne, VIC 3000 Australia; 2 School of Public Health, The University of Hong Kong, Hong Kong, Hong Kong SAR, China; 3 Baker IDI Heart and Diabetes Institute, Melbourne, VIC Australia; 4 Department of Geography, Faculty of Social Sciences, The University of Hong Kong, Hong Kong, Hong Kong SAR, China; 5 Department of Sports Science and Physical Education, Faculty of Education, The Chinese University of Hong Kong, Hong Kong, Hong Kong SAR, China; 6 Elderly Health Service, Department of Health, The Government of Hong Kong SAR, Hong Kong, Hong Kong SAR, China

**Keywords:** Built environment, Walkability, Walking for transport and recreation, Older adults, Mediation analysis, High-density environment

## Abstract

**Background:**

Population growth, population ageing, and urbanisation are major global demographic trends that call for an examination of the impact of urban densification on older adults’ health-enhancing behaviours, such as walking. No studies have examined the pathways through which urban densification may affect older adults’ walking. This information is key to evidence-based, health-oriented urban and transport planning. This study aimed to identify neighbourhood environment characteristics potentially responsible for the effects of neighbourhood densification on older adults’ frequency and amount of transportation and recreation walking within and outside the neighbourhood.

**Methods:**

The Active Lifestyle and the Environment in Chinese Seniors (ALECS) project collected self-reported data from 909 older adults (≥ 65 years) living in 128 physically and socially diverse neighbourhoods in Hong Kong (71% response rate). Walking was measured using the Neighbourhood Walking Questionnaire for Chinese Seniors. Objective residential density and other neighbourhood environmental attributes were assessed using Geographic Information Systems. Generalised additive mixed models examined the total effects of neighbourhood residential density on walking and the mediating role of other environmental attributes and car ownership.

**Results:**

A complex network of potential pathways of positive and negative influences of neighbourhood residential density on different aspects of walking was revealed. While residential density was positively related to within-neighbourhood transportation and outside-neighbourhood recreation walking only, it exhibited positive and/or negative nonlinear indirect effects on all examined aspects of walking via recreation, public transport, food/retail and street intersection densities, and/or car ownership.

**Conclusions:**

High-density environments appear to support within-neighbourhood walking by providing access to food and retail outlets via well-connected street networks and discouraging car ownership. However, extreme density may lead to reductions in walking. Public transport density accompanying high-density areas may facilitate outside-neighbourhood walking but deter within-neighbourhood walking. The development of activity-friendly communities for ageing populations need to consider these opposing influences.

## Background

Population growth, population ageing, and urbanisation are major global demographic trends. The number and proportion of older persons [1] and urban dwellers [2] are increasing in virtually every country across the globe. In 2017, the United Nations reported over 960 million people aged 60 years or over worldwide, corresponding to more than twice the number recorded in 1980, and less than half the number expected in 2050 [1]. Nearly 60% of the global population of older adults live in cities [3], and the transition from rural to urban places is projected to continue [2]. These demographic trends call for an examination of the impact of urbanisation and urban densification on the health and well-being of older populations.

There is growing evidence that the urban built environment plays a key role in shaping older adults’ physical activity behaviour, particularly walking [4–6], which is a key contributor to healthy ageing [7]. Characteristics of the neighbourhood built environment are especially important to older residents whose independence and social contacts can be greatly limited by a poorly-designed community [5, 8–10]. A recent systematic review reported very strong evidence of a positive association between urban densification, operationalised as neighbourhood population or residential density, and older adults’ transportation walking [5]. In contrast, weaker or insufficient support for a positive association was found in relation to overall walking [4] and recreation walking [6].

**Fig. 1:**
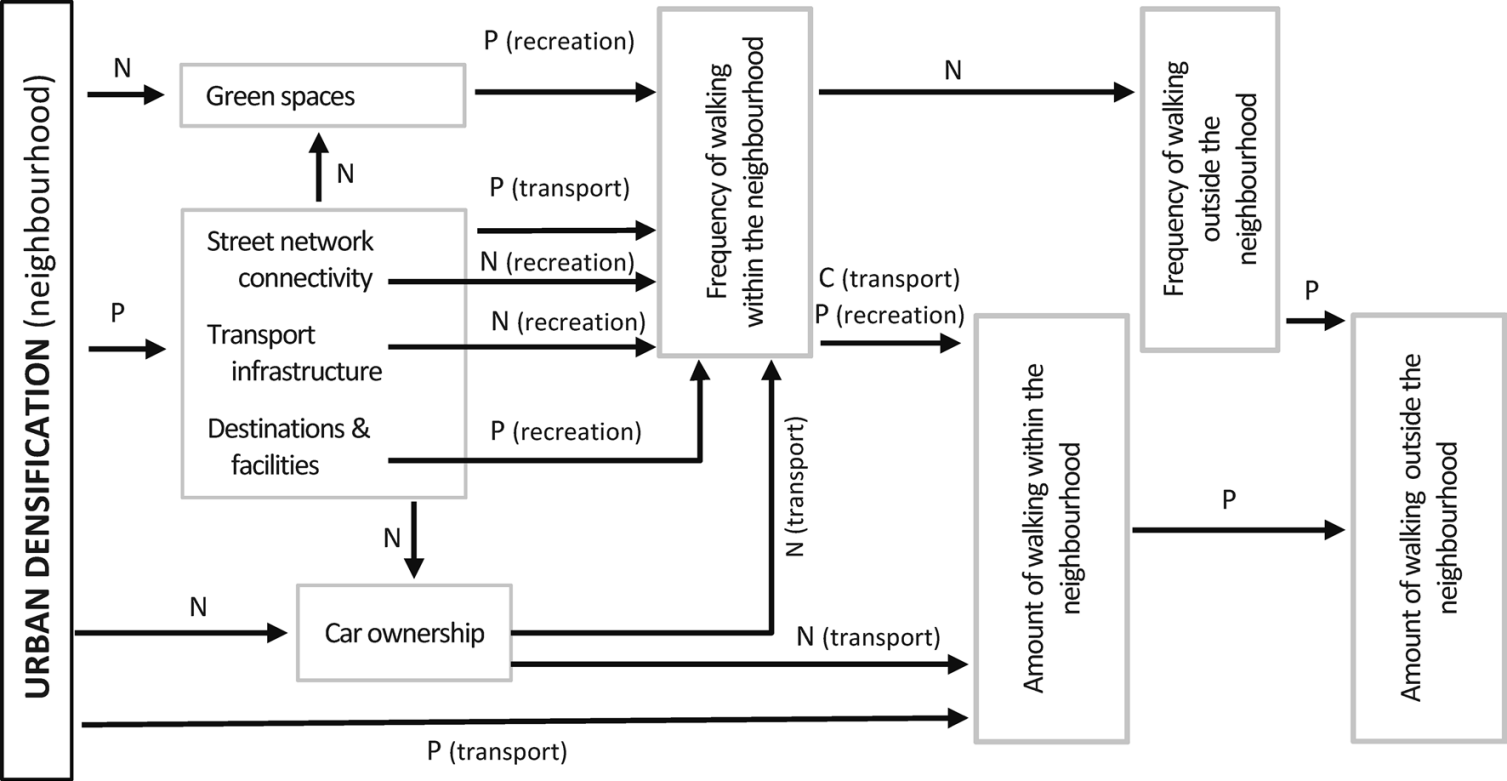
Conceptual model of the effects of urban densification on walking for different purposes. This represents a simplified model of the effects of urban densification on transportation walking and leisure. N, P and C indicate, respectively, hypothesised negative, positive and curvilinear relationships between variables; (transport) and (recreation) indicate that a relationship applies to a specific walking purpose

Despite the substantial number of studies investigating the impact of urban densification on older adults’ walking, the pathways responsible for the observed associations have not been systematically examined. Increases in population and residential density typically result in the development of pedestrian and transportation networks, better accessibility of a wide range of amenities, reductions in the natural environment and increases in motorised traffic [11, 12] (Fig. 1). While some sequels of urban densification (e.g., access to shops) may act as facilitators of walking [5, 13], others (e.g., reductions in green spaces) may act as deterrents [4, 14]. Also, the same sequel may be an enabler as well as a deterrent of walking. For example, while high levels of street intersection density may stimulate transportation walking by providing alternative routes between destinations [12], they may act as a barrier to recreation walking due to the higher levels of traffic-related noise and pollution found in such locations [15]. To further complicate the issue, the magnitude, direction and pathways of influence of urban densification on walking are likely to depend on the purpose (transportation vs. recreation), dimension (frequency vs. amount) and geographical context (within vs. outside the neighbourhood) of walking [4–6]. For example, high residential density may facilitate transportation walking in the neighbourhood via the provision of a variety of destinations of daily living [5] discourage engagement in recreation walking within the neighbourhood due to the lack of green spaces and high levels of traffic [6], and support recreation walking outside the neighbourhood by providing good access to public transport. At higher levels of urban density, increases in neighbourhood residential density may lead to increases in frequency, but reductions in total amount, of transportation walking due to key destinations of daily living being within very short distance from home [16, 17].

Given that densification is the main driver of changes in the urban environment globally, an understanding of the pathways through which it affects various aspects of older adults’ walking behaviour is key to evidence-based, health-oriented urban and transport planning, and community-based walking intervention programs. The distinction between the beneficial and harmful impacts of urban densification on walking is necessary for the development of strategies that enhance beneficial, and eliminate or mitigate the effects of harmful, built environment characteristics resulting from densification. This type of conceptual and analytical approach has the additional advantage of providing less biased estimates of the effects of urban densification and its sequels on walking. Most studies in this research field have examined the effects of single or multiple environmental characteristics on walking without considering whether a characteristic might have lain on the causal pathway from another environmental characteristic to walking [5, 6]. While a regression model with urban densification as the only environmental predictor of walking may provide unbiased estimates of the total effects of urban densification, single variable models of other environmental characteristics shown in Fig. 1 do not because their effects are confounded by urban densification. Further, results of regression models that include urban densification and its sequels (e.g., destination and/or intersection density) as predictors are often misinterpreted as providing estimates of the total independent effects of each environmental predictor, while they only quantify the direct (rather than total) effect of urban densification that is not mediated by its sequels. To address these knowledge gaps, this study aimed to identify neighbourhood built environment characteristics potentially responsible for the effects of neighbourhood densification on older adults’ frequency and amount of transportation and recreation walking within and outside the neighbourhood, following a mediation analytical framework depicted in Fig. 1.

To achieve the proposed aims, we used data from a population-based epidemiological study of environmental correlates of older adults’ physical activity and well-being conducted in Hong Kong. Hong Kong is a particularly relevant location for studying the impact of urban densification on walking behaviours for several reasons. First, Hong Kong has a high level of variation in residential density and related environmental characteristics, enabling a robust assessment of the dose–response relationships between the exposures of interest and walking. In a study examining 15 cities across 11 countries, Hong Kong had the largest variation in residential density and walkability (a composite measure encompassing residential density, street intersection density and land use mix) [18]. For example, while the standard deviation of residential density in Hong Kong was 27,867 dwellings/km^2^, those in Seattle, USA and Ghent, Belgium were 3687 and 6829 dwellings/km^2^, respectively. Another advantage associated with studying the effects of densification on older adults’ walking in Hong Kong pertains to the fact that Hong Kong is an Asian metropolis and Asia is home to nearly 60% of the global population of older adults [1], 50% of whom live in urban areas [3]. Of all continents, Asia has by far the largest number of megacities and one of the most rapid urban growth rates, which is evidenced by a nine-fold increase in urban population between 1950 and 2018 [19]. Finally, international studies on the environment and adults’ physical activity that used common protocols and had Hong Kong as one of the study sites have shown that the environment-physical activity associations observed in Hong Kong were similar to those in Australia, Europe and the Americas [20–22]. This speaks in favour of the international relevance of this study.

## Methods

### Study design

The Active Lifestyle and the Environment in Chinese Seniors (ALECS) study is an observational epidemiologic cross-sectional study conducted in Hong Kong (2012–2016) aiming to examine the relationships of neighbourhood environmental characteristics and psychosocial factors with physical activity, depressive symptoms and quality of life in older community dwellers [23].

ALECS adopted a two-stage sampling strategy whereby participants were recruited from pre-selected neighbourhoods stratified by transport-related walkability and socioeconomic status (SES). This type of design was chosen to maximise the variability in neighbourhood physical environmental attributes related to walking [5] and control for neighbourhood-level socioeconomic differences [24]. To select neighbourhoods, a walkability index was determined for each Tertiary Planning Unit (TPU), the smallest administrative unit with publicly available census data in Hong Kong [25]. The neighbourhood walkability index was a composite measure of TPU-level residential density, street intersection density and land use mix [26, 27], while neighbourhood-level SES was defined as the median household income of a TPU. TPUs were ranked by walkability and SES and those falling into the first four and last four deciles were classified as low and high on the respective neighbourhood characteristic. Walkability and SES scores were crossed to yield four neighbourhood strata: low walkability/low SES, low walkability/high SES, high walkability/low SES, and high walkability/high SES. A total of 124 TPUs were selected, ~ 31 per neighbourhood stratum. Further details on neighbourhood selection have been provided elsewhere [23, 28].

### Participant recruitment

Ethics approval for the conduct of the ALECS study was received from the University of Hong Kong Human Research Ethics Committee for Non-Clinical Faculties and the Department of Health (HKSAR). Hong Kong’s Personal Data (Privacy) Ordinance restricts direct access to contact details of potential participants. Hence, 909 older adults (3–15 participants per TPU) were recruited in person. Recruitment sites included 11 Elderly Health Centres (EHCs) of the Department of Health (72% of the sample) and eight elderly community centres (28% of the sample) located in the selected TPUs. Both EHCs and elderly community centres covered all four TPU strata. The EHCs are distributed across the whole Hong Kong territory to provide primary care services for Hong Kong residents aged 65 years and over. Most participants were recruited from the EHCs because of their willingness to partake in health-related studies endorsed by the Department of Health and the ability of EHC staff to pre-screen participants for health-related study eligibility criteria. Members of the EHCs are generally representative of the elderly population of Hong Kong with respect to age, SES and hospital usage [29]. However, they tend to be more health conscious [30] and, therefore, likely to be more physically active than the general population. For this reason, to examine possible physical activity biases, 28% of participants were recruited from elderly community centres with no provision of medical and health services. Preliminary analyses indicated that participants recruited from the EHCs and community centres did not differ in socio-demographics, health-related variables and walking outcomes.

Potential participants attending a selected EHC or community centre were approached by research staff and invited to partake in the study after determining their eligibility. Inclusion criteria were being 65+ years of age, cognitively intact, able to communicate verbally in Cantonese and walk unassisted for 10+ metres and having lived in one of the selected TPUs for at least 6 months as a community dweller. Eligible participants provided written consent for participation in the study and research staff scheduled a face-to-face interview for data collection. Participants received a HK$50 incentive upon completion of the interview. The study response rate was 71%. Women, participants recruited via community centres and those living in more walkable neighbourhoods were more likely to consent to participating in the study (all *p*s < 0.001). Sample characteristics are presented in Table 1.

**Table 1:** Sample characteristics (N = 909)

Characteristics	Statistics	Characteristics	Statistics
Socio-demographic and health-related characteristics			
Age, years, M ± SD	76.5 ± 6.0	Sex, female, %	66.3
Educational attainment, %		Housing type, %	
Up to primary	56.3	Public and aided	43.1
Secondary or higher	43.7	Private (purchased)	51.3
Marital status, %		Rental	5.6
Married or cohabiting	59.5	Living alone, %	23.1
Widowed	32.7	Household with car, %	28.5
Other	7.8	Number of chronic health problems, M ± SD	3.2 ± 2.0
TPU-level SES, high, %	49.7		
Walking outcomes, M ± SD			
Transportation walking		Recreation walking	
Frequency (times/week)—within neighbourhood	8.1 ± 7.7	Frequency (times/week)—within neighbourhood	3.0 ± 4.0
Amount (min/week)—within neighbourhood	168.7 ± 205.5	Amount (min/week)—within neighbourhood	137.5 ± 220.2
Frequency (times/week)—outside neighbourhood	2.3 ± 4.3	Frequency (times/week)—outside neighbourhood	0.5 ± 1.8
Amount (min/week)—outside neighbourhood	76.1 ± 170.8	Amount (min/week)—outside neighbourhood	32.5 ± 120.1
Neighbourhood environmental attributes (800 m-radius street-network buffers), M ± SD			
Residential density (dwellings/km^2^)	14,295 ± 8444	Street intersection density (intersections/km^2^)	91.5 ± 40.0
Civic and institutional destination density (destinations/km^2^)	69.7 ± 36.5	Entertainment density (destinations/km^2^)	6.9 ± 5.2
Recreation density (destinations/km^2^)	22.5 ± 15.2	Food and retail density (destinations/km^2^)	63.6 ± 37.7
Public transport density (points/km^2^)	11.6 ± 8.5	Park area (hectares)	1.1 ± 1.7

*M* mean, *SD* standard deviation

## Measures

### Neighbourhood environmental characteristics

A neighbourhood was defined as an 800-m street-network buffer surrounding a participant’s residential address, corresponding to an actual 15–20 min walk in any direction among Hong Kong older adults able to walk unassisted [31]. Geographic Information Systems (GIS) data (year: 2011) were used to quantify neighbourhood environmental characteristics. ArcGIS (version 10.3; ESRI) GIS software was used for all spatial analyses. Neighbourhood residential density (dwellings/km^2^) was used as a measure of urban densification and based on data provided by the Census and Statistics Department. Street intersection density was defined as the number of pedestrian-accessible ≥ 3-arm intersections divided by the neighbourhood area (participant’s 800-m residential buffer) and expressed as intersections per km^2^. Centreline road network data sourced from the Lands Department were used to quantify this environmental attribute. Densities of different categories of destinations (food outlets and retail; civic and institutional; entertainment; recreational) and public transport points were derived using data from the Lands and Transportation Departments, respectively. Details on these destinations have been provided elsewhere [32]. Finally, data from the Lands Department were used to compute the total area (in hectares) of public parks within participants’ 800-m street-network residential buffers. Further details about the data sources and spatial resolution for each environmental variable are reported in Additional file 1: Table S1.

### Walking for different purposes

Weekly frequency and minutes of within- and outside-neighbourhood walking for different purposes were assessed using the interviewer-administered Neighbourhood Walking Questionnaire for Chinese Seniors (NWQ-CS) [33]. Participants were asked to report separately frequency and amount (total minutes) of walking within and outside their neighbourhood (defined as an area ~ 15-minute walk from home) for transport and recreation in the usual week. The NWQ-CS was derived from the walking section of the Neighbourhood Physical Activity Questionnaire [34] and adapted for Chinese-speaking older adults. The NWQ-CS has shown good to excellent reliability for all frequency measures (intraclass correlations, ICCs: 0.67 to 0.79) and moderate to good reliability for three of four amount estimates (ICCs: 0.54 to 0.68) [33]. The associations between estimates of walking derived from the NWQ-CS and diaries of walks were moderate to strong (*r*s: 0.41 to 0.90), indicating acceptable levels of measurement validity.

### Sociodemographic and health characteristics

Information on participants’ age, sex, educational attainment, marital status (married or cohabiting, widowed or other), living arrangements (living alone vs. living with others), type of housing (public, private or rental) and household car ownership (household with car vs. household without a car) was collected using an interviewer-administered questionnaire. Educational attainment was recoded to indicate ‘up to primary education’ and ‘at least secondary education’. The number of diagnosed chronic health conditions was determined using information from clinical health-problem checklists compiled by EHC medical staff or participants for those recruited at elderly community centres.

### Data analyses and hypotheses

Descriptive statistics were computed for all variables. As survey data were collected via face-to-face interviews and all participants’ residential addresses could be geocoded, there were no missing data in this study.

The aim of this study was to identify neighbourhood built environment characteristics potentially responsible for the effects of urban densification on older adults’ frequency and amount of transportation and recreation walking within and outside the neighbourhood. Directed acyclic graphs (DAGs) were used to inform mediation analyses and the selection of a minimal sufficient set of confounders of exposure-outcomes, exposure-mediators and mediators-outcomes relationships (Additional file 1: Figures S1, S2 for covariates and hypothesised relationships). The associations presented in the DAGs were based on the hypothesised causal effects among the variables according to the published literature and/or the opinion of an expert panel consisting of four researchers. To estimate confounder-adjusted associations of urban densification with walking outcomes and identify potential mediators of these associations, generalized additive mixed models (GAMMs; [35]) were employed. GAMMs can model data with various distributional assumptions, account for dependency in error terms due to TPU-level clustering (participants sampled from selected TPUs) and estimate complex dose–response relationships of unknown form [35]. Curvilinear associations were estimated using smooth terms modelled with thin plate splines [35]. If the data did not provide sufficient evidence of a curvilinear association, smooth terms were replaced by linear terms. Model selection (linear vs. curvilinear effect) was based on Akaike Information Criterion (AIC) values, where a lower AIC was indicative of a better-fitting model. A ≥ 5-unit difference in AIC was used as the criterion for model selection [21, 36]. Potential multicollinearity was assessed by computing the Variance Inflation Factor (VIF) for each variable included in the models. A VIF > 5 was considered to be problematic [37]. Analyses followed several steps detailed in the Supplementary Material and were conducted in R version 3.4.3 [38] using the packages ‘mgcv’ version 1.8.22 [35] and ‘multcomp’ version 1.4.8 [39].

### Total effects of urban densification on walking

The total effects of urban densification (neighbourhood residential density) on each of the eight walking outcomes (e.g., weekly frequency of within-neighbourhood transportation walking) were first estimated (Step 1 in Additional file 1: Table S1). These GAMMs were adjusted for potential confounders, i.e., factors potentially associated with neighbourhood self-selection (choosing to live in low- or high-density areas) and walking outcomes (see Additional file 1 for details). Given that the allocation of public housing in Hong Kong is not based on individual preferences for residential location [40], type of housing (public, private and rental housing) was considered as a moderator of densification-walking associations to examine the potential effect of neighbourhood self-selection.

It was hypothesised that neighbourhood residential density would be positively related to transportation walking within the neighbourhood [5] but unrelated to transportation walking outside the neighbourhood. Nil rather than negative associations between residential density and transportation walking outside the neighbourhood were expected because low-density areas typically have poorer public transport services than high-density areas [41]. Namely, although older residents of low-density neighbourhoods may need to visit other areas for various activities, they may do it relatively infrequently as public transport options may be limited. On the other hand, high levels of public transport availability, accessibility and connectivity may motivate older residents in high-density areas to take discretionary trips outside their neighbourhood. While residential density was hypothesised to be unrelated to within-neighbourhood recreation walking [6], positive associations were expected between residential density and recreation walking outside the neighbourhood because parks, beach/river waterfronts and walking trails have been identified among the most popular destinations for recreation walking [42, 43] and these destinations are typically located in low-density areas [44].

### Mediated and direct effects of urban densification on walking

The presence of mediation effects was examined using the joint-significance test [45] and following the steps outlined in Additional file 1: Table S1. According to this test, mediation is confirmed if the associations (regression coefficients) between an exposure and its mediator(s), and the exposure-adjusted associations between the mediator(s) and the outcome are statistically significant. It was hypothesised that higher residential density would lead to the development of a more interconnected street network and increases in public transport and destination densities (Fig. 1) [46]. Higher residential, public transport and destination densities were expected to result in less green space (park area) [44] and household car ownership to be more prevalent among respondents living in areas with lower residential, street intersection, food/retail, recreation and public transport densities [47]. Frequency of within-neighbourhood transportation walking was expected to be negatively related to household car ownership [48] and positively associated with street intersection and destination densities [5]. In contrast, street intersection and public transport densities were expected to be negatively related to frequency of recreation walking within the neighbourhood because they are associated with higher levels of noise and pollution [49], which have been found to deter recreation walking in Hong Kong older adults [50]. We also hypothesised that park area [43, 50] and recreation density [51] would be positively associated with this walking measure.

Frequency of within-neighbourhood walking was expected to partially mediate the effects of environmental attributes on the amount of within-neighbourhood walking because, for example, having more shopping/retail destinations nearby may not only result in a greater number of utilitarian trips but also in the visitation of a greater number of shops per trip and, hence, more walking. Similarly, having larger rather than smaller parks in the area may motivate more frequent and longer walks for recreation. Frequency of within-neighbourhood walking was expected to be negatively related to frequency of outside-neighbourhood walking and partially mediate/supress the effects of dwelling density via public transport density. The effects of environmental attributes on amount of walking outside the neighbourhood were expected to be fully mediated by the other domain-specific walking variables.

## Results

On average, participants walked more frequently for transportation than recreation, which resulted in them accumulating greater amounts (min/week) of transportation than recreation walking (Table 1). However, the average walking trip duration was greater for recreation (45.8 min/trip within the neighbourhood; 65.0 min/trip outside the neighbourhood) than transportation purposes (20.8 min/trip within the neighbourhood; 33.1 min/trip outside the neighbourhood). Fewer trips were undertaken outside than within the neighbourhood (Table 1). Consequently, the amount of walking accrued outside the neighbourhood was also smaller than that accumulated within the neighbourhood (Table 1), despite the average trip duration being longer for outside- than within-neighbourhood walks, as noted above. Participants resided in neighbourhoods with substantial differences in all examined environmental attributes as evidenced by the means and standard deviations reported in Table 1, yielding coefficients of variations ranging from 0.44 to 1.54.

The VIFs for the variables included in the analyses ranged from 1.04 (household car ownership) to 3.54 (civic and institutional destination density), with mean and median values of 1.90 and 1.71, respectively. Given that all VIF values were substantially lower than 5 [37], no multicollinearity issues were identified.

### Total effects of densification on walking

Table 2 reports the total effects of neighbourhood residential density on the eight walking measures. Neighbourhood residential density was positively associated with both measures of within-neighbourhood transportation walking and unrelated to both measures of outside-neighbourhood transportation walking. The opposite held true for recreation walking, with only measures of outside-neighbourhood walking being positively related to neighbourhood residential density.

**Table 2:** Total effects (associations) of neighbourhood residential density (1000 units/km^2^) on walking

	Transportation walking	Recreation walking
***Walking measure***	e^*b*^ (95% CI)	e^*b*^ (95% CI)
Frequency (times/week)—within neighbourhood	1.008 (1.001, 1.015)	1.002 (0.992, 1.012)
Amount (min/week)—within neighbourhood	1.015 (1.005, 1.025)	1.004 (0.987, 1.021)
Frequency (times/week)—outside neighbourhood	1.003 (0.992, 1.015)	1.014 (1.005, 1.024)
Amount (min/week)—outside neighbourhood	0.997 (0.979, 1.016)	1.053 (1.021, 1.087)

Models were adjusted for covariates listed in Additional file 1: Table S2—model 1T

*e*^*b*^ exponentiated regression coefficient, *CI* confidence intervals

### Direct and mediated effects of densification on walking

#### Transportation walking

**Fig. 2:**
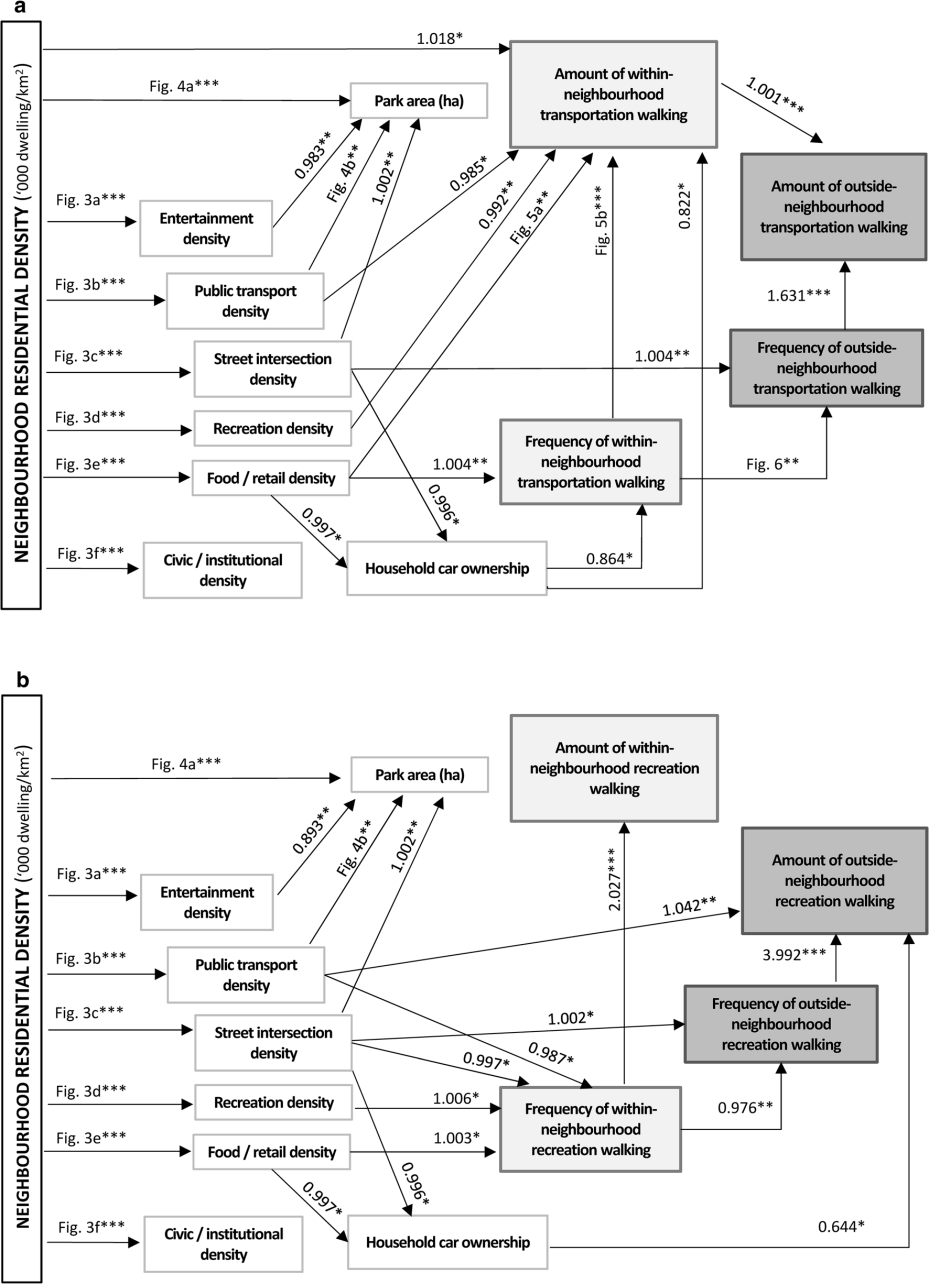
Direct and indirect effects of residential density on walking for transport (**a**) and recreation (**b**). Only significant associations (*p *< 0.05) are reported. Values represent exponentiated regression coefficients. For example, 1.631 in panel **a** indicates a 63.1% increase in amount of outside-neighbourhood transportation walking followed by a 1 unit increase in frequency of outside-neighbourhood transportation walking. Significant curvilinear associations between pairs of variables are labelled by the figure number depicting them (e.g., Fig. 3**a** referring to Fig. 3—panel **a**, representing the association between neighbourhood residential density and entertainment density). Point estimates and confidence intervals of all examined associations are reported in Additional file 1: Tables S1–S7. * *p *< 0.05; ** *p *< 0.01; *** *p *< 0.001

**Fig. 3:**
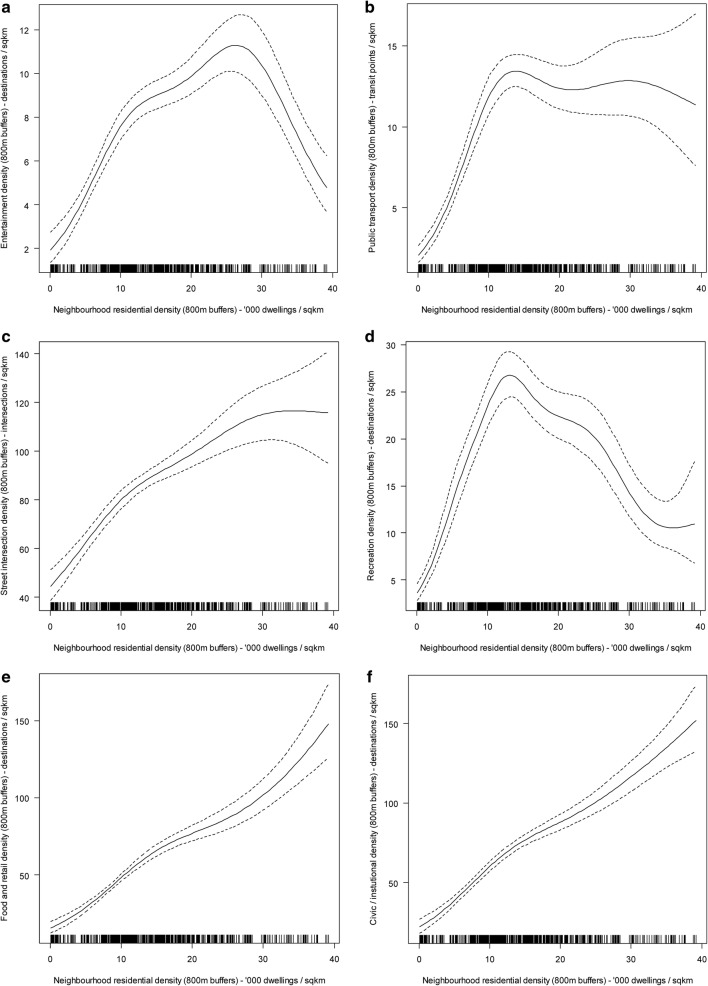
Nonlinear relationships of residential density with other environmental attributes. **a** Recreation density; **b** public transport density; **c** street intersection density; **d** recreation density; **e** food and retail density; **f** civic/institutional density. The solid lines represent point estimates and the dotted lines represent the 95% confidence intervals of the point estimates

Panel a of Fig. 2 summarises the findings of the mediation analyses for transportation walking. Detailed model outputs (point estimates, 95% CI and *p*-values for all regression coefficients) are presented in Additional file 1: Tables S2–S8. The positive effects of neighbourhood residential density on frequency of within-neighbourhood transportation walking were fully mediated by food/retail density, street intersection density and household car ownership (Fig. 2; panel a). Specifically, positive curvilinear effects of residential density on food/retail (Fig. 3; panel e) and intersection densities (Fig. 3; panel c) were observed. These two environmental attributes were negatively associated with the likelihood of having a car in the household (food/retail: e^*b*^ = 0.997; 95% CI 0.993, 0.999; street intersection: e^*b*^ = 0.996; 95% CI 0.993, 0.999) which, in turn, was negatively associated with frequency of within-neighbourhood walking (e^*b*^ = 0.864; 95% CI 0.765, 0.976). In addition, a positive direct effect of food/retail density (unmediated by car ownership) was observed on frequency of within-neighbourhood walking for transport.

**Fig. 4:**
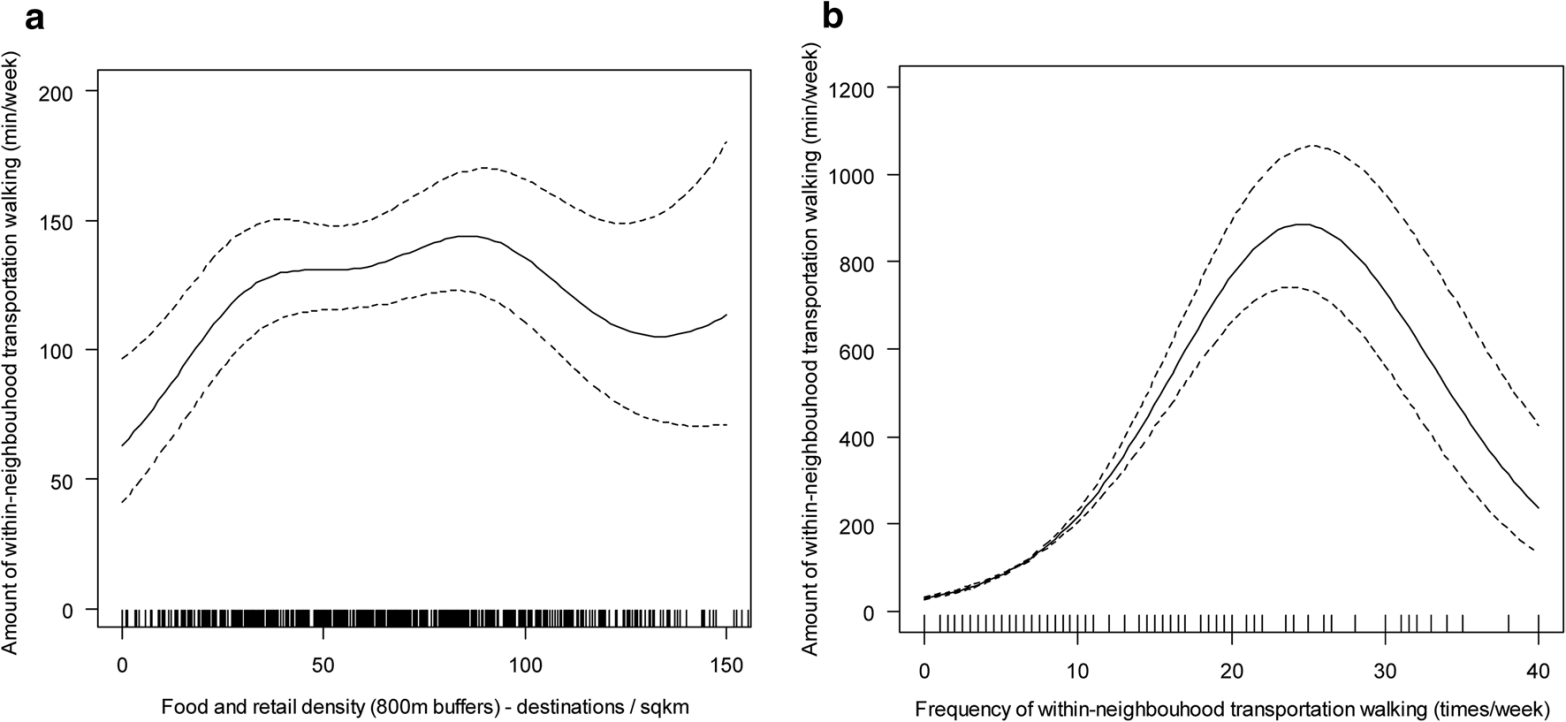
Relationships of food/retail density (**a**) and frequency of within-neighbourhood transportation walking (**b**) with amount of within-neighbourhood transportation walking. The solid lines represent point estimates and the dotted lines represent the 95% confidence intervals of the point estimates

A complex network of inconsistent mediators (mediators with opposite effects) explained in part the association between residential density and amount of within-neighbourhood transportation walking (Fig. 2; panel a). Residential density impacted on the amount of within-neighbourhood walking through seven pathways, four of which went through food/retail density. Two of these four pathways involved frequency of within-neighbourhood walking as a mediator, which showed an inverted-U relationship with amount of within-neighbourhood walking (Fig. 4; panel b). Specifically, amount of walking increased with frequency of walking up to 25 walking trips per week, decreased thereafter but remained above the average amount of ~ 170 min/week. One of the other two pathways via food/retail density was mediated by household car ownership, which showed a negative association with amount of within-neighbourhood walking for transport (e^*b*^ = 0.822; 95% CI 0.686, 0.906). The fourth pathway via food/retail density was direct and indicated a curvilinear effect of food/retail density on amount of walking unmediated by car ownership and frequency of walking (Fig. 4; panel a).

Residential density had two more indirect effects on amount of within-neighbourhood walking: one through public transport density, the other through recreation density (Fig. 2; panel a). The former was primarily negative, where residential density exhibited a positive curvilinear association with public transport density (Fig. 3; panel b) which, in turn, was predictive of lower amounts of within-neighbourhood walking for transport (e^*b*^ = 0.985; 95% CI 0.971, 0.998). The indirect effect via recreation density was curvilinear since an inverted-U relationship was observed between residential density and this neighbourhood attribute (Fig. 3; panel d), and a negative association was found between recreation density and amount of within-neighbourhood walking for transport (e^*b*^ = 0.992; 95% CI 0.985, 0.998). Increases in residential density up to ~ 12,000 dwellings/km^2^ were associated with increases in recreation density (Fig. 3; panel d) which had a negative impact on amount of walking. In contrast, increases in residential density above that threshold were associated with a decrease in recreation density accompanied by an increase in amount of walking. Finally, the positive effects of residential density on amount of within-neighbourhood transportation walking were not fully mediated by environmental attributes, household car ownership and frequency of walking. After accounting for all these variables, a positive, but weaker, association remained (e^*b*^= 1.018; 95% CI 1.003, 1.033).

**Fig. 5:**
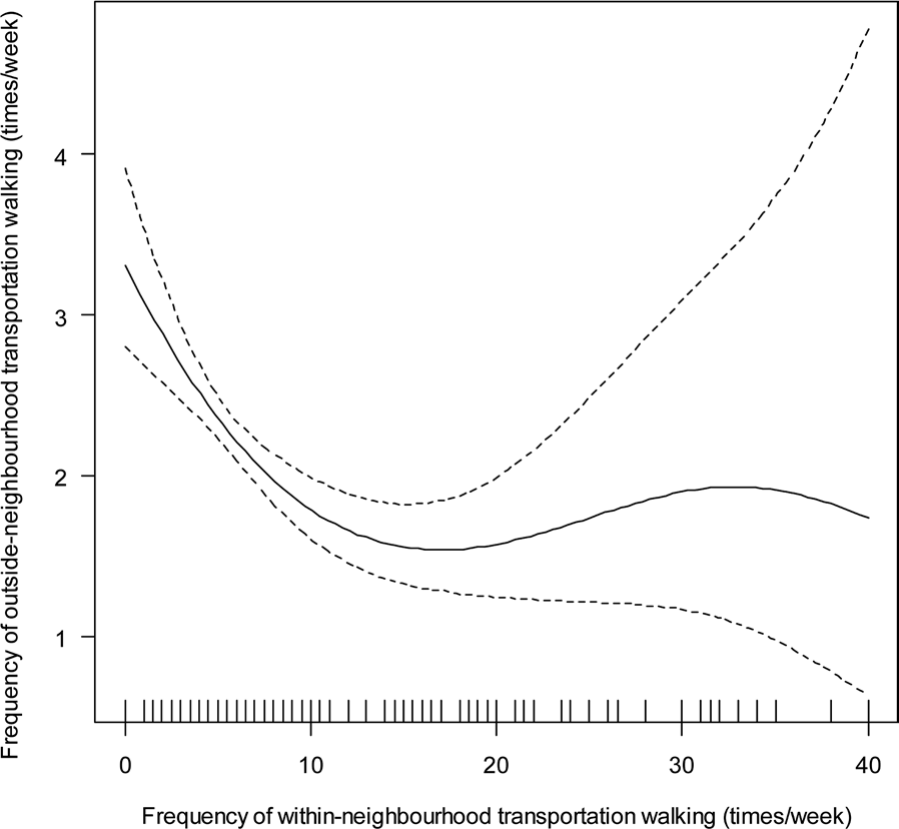
Relationship between frequency of within-neighbourhood and outside-neighbourhood transportation walking. The solid line represents point estimates and the dotted lines represent the 95% confidence intervals of the point estimates

Although the total effect of residential density on outside-neighbourhood walking for transport was not significant (Table 2), significant indirect effects were identified (Fig. 2; panel a). A positive indirect effect on frequency of outside-neighbourhood walking via street intersection density was found (e^*b*^ = 1.004; 95% CI 1.001, 1.007). A nonmonotonic curvilinear direct effect on frequency of outside-neighbourhood transportation walking through frequency of within-neighbourhood walking (and its correlates) was also observed. Increases up to ~ 17 trips per week in frequency of within-neighbourhood transportation walking were associated with a decrease in frequency of outside-neighbourhood walking, which plateaued thereafter (Fig. 5). Finally, neighbourhood residential density showed indirect effects on the amount of outside-neighbourhood transportation walking through frequency of outside-neighbourhood (e^*b*^= 1.631; 95% CI 1.585, 1.679) and amount of within-neighbourhood transportation walking (e^*b*^= 1.001; 95% CI 1.001, 1.002) and their mediators.

**Fig. 6:**
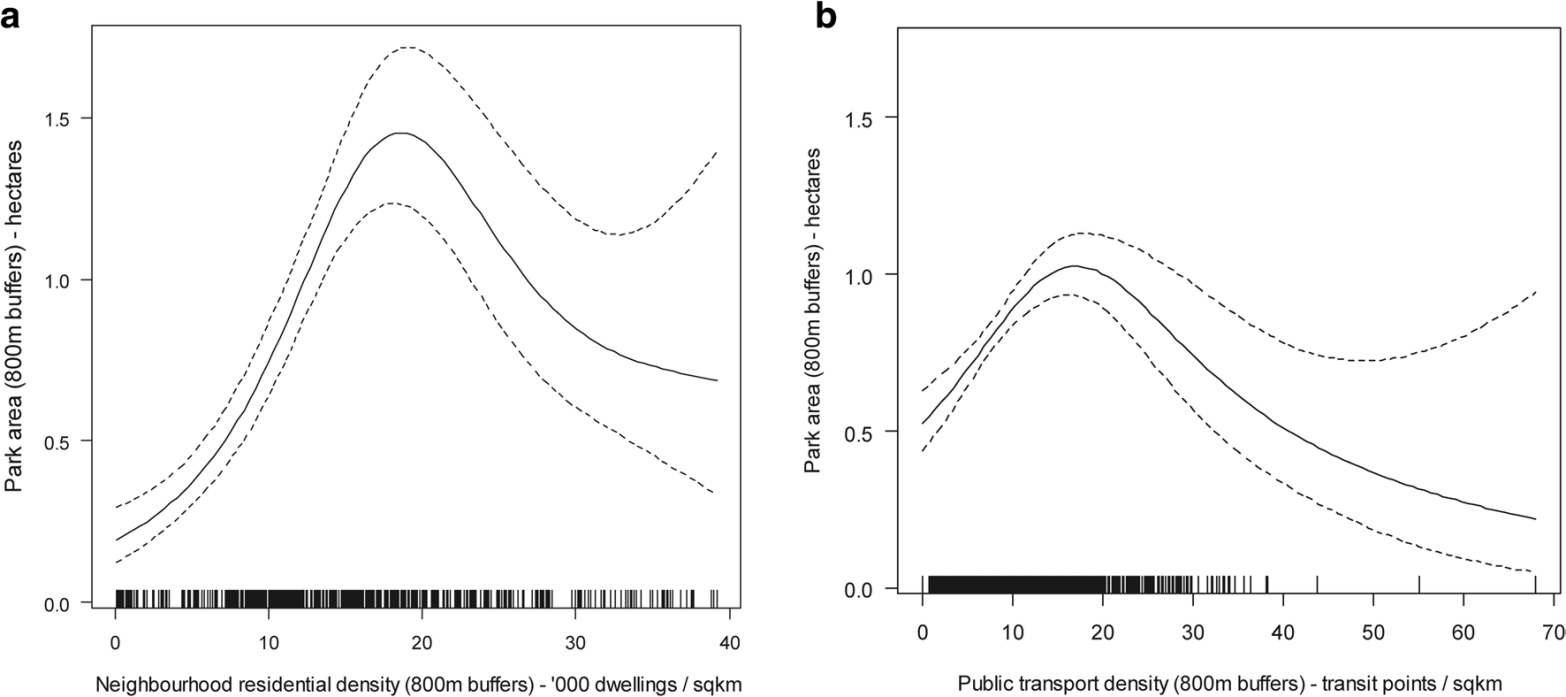
Nonlinear relationships of residential (**a**) and public transport (**b**) density with park area. The solid lines represent point estimates and the dotted lines represent the 95% confidence intervals of the point estimates

Park area and civic and institutional destination density were not identified as significant mediators of residential density-transportation walking associations (Fig. 2; panel a), although they were related to residential density (Fig. 3; panel f; Fig. 6), and park area was also related to entertainment (e^*b*^= 0.983; 95% CI 0.973, 0.993), public transport (Fig. 6; panel b) and street intersection densities (e^*b*^= 1.002; 95% CI 1.001, 1.004).

### Recreation walking

Panel b of Fig. 2 summarises the findings of the mediation analyses for recreation walking. Detailed outputs are provided in Additional file 1: Tables S2–S8. Although the total effects of neighbourhood residential density on within-neighbourhood recreation walking were not significant, significant indirect effects through other environmental variables were found (Fig. 2; panel b). Two negative and two positive indirect effects on frequency of within-neighbourhood recreation walking emerged. Residential density had negative effects through public transport (e^*b*^= 0.987; 95% CI 0.976, 0.998) and street intersection densities (e^*b*^= 0.997; 95% CI 0.995, 0.999) and a positive effect through food/retail density (e^*b*^= 1.003; 95% CI 1.000, 1.006). The indirect effect via recreation density was nonmonotonic curvilinear, wherein increases in residential density up to ~ 12,000 dwellings/km^2^ were associated with increases in recreation density (Fig. 3; panel d) which, in turn, were associated with increases in frequency of within-neighbourhood recreation walking (e^*b*^= 1.006; 95% CI 1.001, 1.011). Conversely, increases in residential density above ~ 12,000 dwellings/km^2^ were associated with decreases in recreation density (Fig. 3; panel d) leading to reductions in frequency of within-neighbourhood walking for recreation.

All indirect effects of residential density on amount of within-neighbourhood recreation walking were channelled through frequency of within-neighbourhood recreation walking, which was strongly linearly related to amount of walking (e^*b*^= 2.027; 95% CI 1.968, 2.087) (Fig. 2; panel b). Frequency of within-neighbourhood recreation walking (and its mediators) was also the pathway through which residential density impacted on frequency of outside-neighbourhood walking (e^*b*^= 0.976; 95% CI 0.960, 0.992): the higher the frequency of walking within the neighbourhood, the lower the frequency of walking outside the neighbourhood. In addition, residential density had a positive effect on frequency of outside-neighbourhood walking through street intersection density (e^*b*^= 1.002; 95% CI 1.000, 1.004). These mediators explained in full the positive association between residential density and frequency of outside-neighbourhood recreation walking.

The total positive effect of residential density on amount of outside-neighbourhood walking for recreation was fully accounted for by eight pathways, five of which went through frequency of outside-neighbourhood walking (Fig. 2; panel b), which was strongly positively related to amount of outside-neighbourhood walking (e^*b*^= 3.992; 95% CI 3.592, 4.436). In addition, residential density exerted positive indirect effects on this walking outcome via public transport density (e^*b*^= 1.042; 95% CI 1.012, 1.074) and via the effects of street intersection and food/retail densities on household car ownership (Fig. 2; panel b). Higher residential density was curvilinearly associated with higher street intersection and food/retain densities which, in turn, were related to lower odds of car ownership. Not having a car in the household was, in turn, predictive of more weekly minutes of outside-neighbourhood walking for recreation (e^*b*^= 0.644; 95% CI 0.420, 0.988).

To estimate the potential impact of neighbourhood self-selection on the total and mediated effects of residential density on walking, housing type was examined as a moderator of the above associations with household car ownership and walking outcomes. Housing type was not a significant moderator of the total and direct effects of residential density or the direct effects of other environmental attributes [mediators] on walking (Additional file 1: Tables S9–S14).

## Discussion

As a major global demographic trend, urban densification has the potential to impact on the health and well-being of a large proportion of the global population by affecting health-related lifestyle behaviours, such as regular participation in physical activity, and increasing exposure to air and noise pollution and other environmental stressors. This study aimed to elucidate the impact of urban densification on the most common type and one of the most equitable forms of physical activity (i.e., walking) in the fastest growing age group globally (people aged 65 years and over). A complex network of potential pathways of influence on different aspects of walking behaviour was revealed.

Neighbourhood residential density was related to all seven environmental attributes examined in this study, four of which acted as potential pathways of influence of densification on both transportation and recreation walking (Fig. 2). These included street intersection, food and retail, recreation and public transport densities. All these attributes have been previously found to be positively related to either transportation, recreation or total walking in older adults [4–6].

### Transportation walking

Food/retail and street intersection densities were the main environmental features through which residential density impacted on transportation walking. As confirmed in this study, high density neighbourhoods offer access to a large number of food and retail outlets [46], which are the primary motivators of utilitarian walking trips within the neighbourhood [5]. This study also supports the hypothesis that, via the provision of food/retail destinations and an interconnected street network, densification reduces car dependency [47] resulting in more active utilitarian trips and higher amounts of transportation walking in the local community [48].

As expected, by exerting a positive effect on the number of within-neighbourhood transportation walks, residential density and its sequels, in general, contributed to the accumulation of higher amounts of transportation walking in the neighbourhood. However, in older adults who reported more than ~ 25 walks per week, increases in frequency of within-neighbourhood walking were associated with the accumulation of fewer minutes of the same type of walking. This suggests that, in ultra-dense metropolises across the globe, destinations of daily living may be so close to home and one another (i.e., 5-minute walk) that residents can afford visiting them more frequently and/or without resorting to multi-destination trip chaining. If they engage in trip chaining, they likely accumulate fewer minutes of walking because trip segments are shorter [52]. In support of this supposition, a recent study on Hong Kong older adults reported positive associations of destination accessibility with frequency of within-neighbourhood transportation walking but negative associations with amount of the same type of walking in destination-rich areas [16].

The positive impacts of residential density on amount of within-neighbourhood walking for transport were not only channelled through frequency of walking and household car ownership (Fig. 2). A direct effect was also found. Crowdedness and traffic in high density areas may force pedestrians to walk at a slower pace, which would increase the time to reach destinations. High density areas may also host a larger number of interesting and diverse commercial destinations to visit which may increase the total duration of walking trips, as suggested by the primarily positive direct effect of food and retail density on amount of walking. Not all pathways linking residential density to amount of within-neighbourhood transportation walking were positive. Having more public transport and recreational options in the neighbourhood was associated with shorter utilitarian walks. Owing to the low concessionary fares for the elderly in Hong Kong (up to 2 HK$ per trip = 0.26 US$), older residents of areas with high public transport density, which is usually accompanied by a high frequency service, may prefer getting to/from destinations in their neighbourhood via public transport rather than walking because it is more convenient. This is likely to apply to other urban environments globally that provide affordable public transport to ageing populations.

Although previous studies have found positive associations between access to recreational facilities and within-neighbourhood transportation walking [5], they did not adjust for residential density and other environmental attributes, or used recreational destination diversity [31] rather than density as the exposure. The negative association between this type of destinations and amount of within-neighbourhood transportation walking could be due to several reasons. First, the discretionary nature of recreational activities compared to activities of daily living (e.g., shopping, eating) suggests that recreational destinations are likely to be visited by fewer residents than food/retail outlets [42, 53] and, hence, promote lower average levels of utilitarian walking. Second, trip chaining, which results in longer utilitarian walks, is more common for shopping and errand purposes (entailing visitation of commercial destinations) than recreational purposes [53, 54].

Although, as expected, we did not find sufficient evidence for a total effect of residential density on outside-neighbourhood transportation walking, negative and positive indirect effects were observed. As older adults living in higher density neighbourhoods walked more frequently for transportation within the neighbourhood for the reasons explained above, they needed to walk less frequently for the same purpose outside the neighbourhood. This negative indirect effect was offset by a pathway through street intersection density. Better street connectivity in high density areas appeared to facilitate walking outside the neighbourhood. This is in line with previous findings providing stronger evidence of an association between street connectivity and total rather than within-neighbourhood transportation walking [5]. It is possible that interconnected street networks may make it easier for older adults to walk longer distances or that older adults having to cross more roads en route may tend to define their neighbourhood (15-minute walk from home) as a smaller area than those living in neighbourhoods with fewer crossings. Studies utilising Global Positioning System (GPS) or smartphone technology are needed to clarify this finding as self-report measures of walking within and outside the neighbourhood rely on participants’ perceptions of the duration of walking trips which can be inaccurate [55, 56].

Interestingly, several positive and negative indirect effects of residential density on the amount of outside-neighbourhood transportation walking were channelled through amount of within-neighbourhood walking, which was positively rather than negatively associated with outside-neighbourhood walking (conditional on walking frequency). This positive association could be due to individual differences in walking speed (e.g., older adults walking slowly taking more time to reach destinations within as well as outside the neighbourhood) or the fact that the health benefits and active transportation habits accrued from living in a walkable neighbourhood [10] also result in residents being able or willing to be more active elsewhere.

#### Recreation walking

While, as noted previously [51, 57], neighbourhood residential density was unrelated to within-neighbourhood recreation walking, several significant indirect effects of opposite direction were observed, pointing at possible ways to make high density areas more attractive for recreation walking. In contrast to what a recent systematic review on this topic suggests [6], enhanced access to food, retail and recreational destinations emerged as potential pathways through which urban densification may promote walking for recreation in the local area. Though this type of walking occurs primarily in parks and along the streets [40], the presence of restaurants, cafés or shops along the route is important to older adults who need to rest during their walks [58] or to those who engage in window shopping and social activities as part of their recreational walks [59]. Recreational destinations (e.g., sports fields) are rarely listed as places for recreation walking [42]. However, older adults who visit such destinations to exercise often travel to/from them on foot [53] and they may consider these walks as recreational. It is worth noting that, in this study, recreational destinations tended to decrease with density and, hence, lead to a reduction in walking in areas with more than ~ 12,000 dwellings/km^2^. This suggests that moderate levels of density may be optimal for recreation walking.

Residential density also exerted negative effects on within-neighbourhood recreation walking that cancelled out the positive impacts of food, retail and recreational destinations. Specifically, higher residential density was associated with higher street intersection and public transport densities which were negatively associated with walking frequency. Neighbourhoods with more street intersections and public transport stops may discourage walking for recreation by increasing residents’ exposure to traffic hazards, fumes and noise [60]. In addition to regulating traffic emissions, these negative sequels of densification may be overcome by the constructions of walkaways and bridges allowing pedestrians to avoid direct exposure to trafficked areas [61]. In fact, in an earlier study, the presence of bridges and overpasses was the strongest predictor of Hong Kong older adults’ participation in within-neighbourhood recreation walking [51]. Of course, it would be even better to reduce traffic-related pollution and noise in high-density areas by limiting car traffic on certain roads.

As observed for transportation walking, residents who frequently walked for recreation within the neighbourhood were less likely to walk for the same purpose outside the neighbourhood. Thus, by affecting within-neighbourhood recreation walking through four different pathways, residential density also impacted on recreation walking outside the neighbourhood. Again, similarly to transportation walking, residential density had a positive impact on frequency of outside-neighbourhood recreation walking via street intersection density suggesting that this environmental sequel of densification may facilitate recreational walking outside the neighbourhood by providing shorter routes to such destinations [62] or may influence one’s perceptions of neighbourhood boundaries. In general, previous studies provided more, although tenuous, support for a link between street connectivity and total rather than within-neighbourhood recreation walking [6], which supports the present findings that this attribute may be more important for outside-neighbourhood recreation walking trips.

Public transport density and food/retail density via household car ownership mediated the positive effects of residential density on the amount of outside-neighbourhood recreation walking. Not having a car in the household and living in a neighbourhood with many public transport options predicted longer recreational walks outside the neighbourhood. Having access to more transit routes in the neighbourhood makes it easier to access remote, larger parks and other natural open spaces (e.g., beaches) where older residents can enjoy longer walks. Although it could be argued that this line of reasoning is also applicable to older adults with a car in the household, most older adults in China (including Hong Kong) do not own a car [63, 64] but rely on their usually time-poor adult children to drive them to/from places [65, 66]. This could explain why having a car in the household was associated with shorter recreational walks outside the neighbourhood. Future studies need to establish whether this particular effect differs in countries with a high prevalence of car ownership and driving among older adults.

It is noteworthy that although previous research reported positive associations between parks and recreation walking in older adults [43, 50], the present study found insufficient evidence of an effect. Also, an unexpected positive association between street intersection density and park area was observed (Fig. 2). Better neighbourhood street connectivity (quantified as street intersection density) may yield larger street-network residential buffers which, in turn, may impact on the amount of park area falling within the buffers and bias the relationships between park area and recreation walking. In fact, post hoc analyses of the relationships between these variables showed that street intersection density was positively associated with buffer size (*r *= 0.36) and buffer size was positively associated with park area (*r *= 0.46). This observation may explain why, in general, the evidence of an association between parks and recreation walking in older adults is tenuous [6]. Future research needs to consider the pro and cons of using various measures of access to parks (e.g., park area, distance to nearest park, number of parks in the buffer, percentage of park area in the buffer) as correlates of walking.

### Practical implications

Important urban planning implications are indicated by the positive and negative impacts of urban densification on older adults’ walking. By strengthening the pathways that encourage walking and minimising or mitigating those that discourage it, we can create cities that can sustainably support healthy and active ageing. It is important to create an environment that primarily supports walking for different purposes within the neighbourhood but also provides opportunities to maintain the same level of life-space mobility across time (the size of the spatial area individuals move through in daily life and the frequency) [67]. Older adults typically wish to continue living at home in their local community as they age [68]. This relies on them maintaining their physical capacity to walk outdoors for daily activities, which, in turn, relies on having services, amenities and activity facilities close to home and easy-to-walk routes to reach such destinations [69–71].

Overall, this study suggests that, within the context of an ultra-dense metropolis, moderate-to-high levels of urban density (10–25,000 dwellings/km^2^) may be optimal to promote walking in older adults. Extreme levels of density are associated with higher frequency but smaller amounts of walking for transport within the neighbourhood; fewer walking trips outside the neighbourhood limiting life-space mobility; no further increases in access to public transport and street connectivity and, hence, no further disincentives to car ownership or support for walking outside the neighbourhood; and fewer recreational facilities leading to less walking for recreation in the neighbourhood. To promote walking for different purposes among older adults, urban densification should be accompanied by the provision of a variety of food, retail and recreational facilities, an interconnected network of paths and elevated walkaways suited to pedestrians, and accessible public transport corridors.

## Limitations

The cross-sectional nature of the study limits evidence of causality that might support policy change. Walking for different purposes within and outside the neighbourhood was assessed using a validated self-report measure that tends to underestimate the amount of walking for transport [33]. However, no other validated tools to assess location- and purpose-specific walking were available. Future studies should use more accurate and reliable methods to identify the locations, frequency, duration and purpose of walks. GPS devices combined with walking diaries have been suggested for this purpose [53]. However, the built environment in ultra-dense metropolises like Hong Kong poses significant challenges to GPS data collection because the GPS signal is often blocked by high-rise buildings and indoor, covered walkaways [72]. Alternative methods appropriate for older adults and any type of urban environment include map-based interviews [73, 74] and smartphone technologies combined with ecological momentary assessments [75].

This study used a sampling strategy designed to maximise the variability of environmental exposures and was limited to older adults able to walk without assistance for 10+ metres, yielding a sample not representative of the general population of older adults. However, the aim of this study was to examine environment-walking associations rather than obtain population estimates of walking and environmental exposures. Park area within street-network residential buffers may not be an optimal measure of relative exposure to green spaces because it is in part determined by street connectivity. The pattern of associations between urban density, other environmental attributes and walking observed in this study may be different to those in cities with different topography, climate, transportation system and household car ownership level. There is, therefore, a need for similar studies in other geographical contexts.

## Conclusions

This study has demonstrated the importance of examining the various pathways through which urban densification may impact on older adults’ walking. An intricate network of contrasting linear and curvilinear pathways of influence were found suggesting that high density environments support walking within the neighbourhood by providing access to many food and retail options via a network of well-connected streets which, in turn, discourage car ownership. However, extreme levels of density and destination accessibility appear to lead to reductions in transportation walking and recreational facilities, which are an important incentive for recreation walking in the local area. This study also suggests that street connectivity accompanying high density areas facilitates walking outside the neighbourhood but deters walking for recreation within the neighbourhood and, similarly, public transport density facilitates walking for recreation outside the neighbourhood but deters walking within the neighbourhood. This study highlights the importance of examining separately the contextual and behavioural aspects of walking for different purposes and the potential causal pathways linking built environmental features to gain a better understanding of how the neighbourhood built environment impacts on walking behaviour.

## Supplementary information

**Additional file 1: Table S1.** Definitions of environmental variables (exposures), data sources and spatial resolution. **Table S1.** Outline of regression analyses. **Table S2.** Models 1M: Direct effect of neighbourhood residential density [exposure] on street intersection density, public transport density and density of four destination types [mediators 1]. **Table S3.** Model 2aM: Direct effects of neighbourhood residential density and mediators 1 on park area [mediator 2]. **Table S4.** Model 2bM: Direct effects of neighbourhood residential density and mediators 1 on household car ownership [mediator 2]. **Table S5.** Model 3M: Direct effects of neighbourhood residential density, mediators 1, park area and household car ownership [mediators 2] on frequency of within-neighbourhood walking [outcome]. **Table S6.** Model 4M: Direct effects of neighbourhood residential density, mediators 1, mediators 2 and domain-matching frequency of within-neighbourhood walking [mediator 3] on amount of within-neighbourhood walking [outcome]. **Table S7.** Model 5M: Direct effects of neighbourhood residential density, mediators 1, mediators 2, and mediator 3 on frequency of outside-neighbourhood walking [outcome]. **Table S8.** Model 6M: Direct effects of neighbourhood residential density, mediators 1, mediators 2, mediator 3, domain-matching frequency of outside-neighbourhood walking and amount of within-neighbourhood walking [mediators 4] on amount of outside-neighbourhood walking [outcome]. **Table S9.** Housing type as a moderator of total effects of neighbourhood residential density on walking measures. **Table S10.** Housing type as a moderator of direct effects of neighbourhood residential density and mediators 1 on household car ownership [mediator 2]. **Table S11.** Housing type as a moderator of direct effects of neighbourhood residential density, mediators 1 and park area on frequency of within-neighbourhood walking [outcome]. **Table S12.** Housing type as a moderator of direct effects of neighbourhood residential density, mediators 1 and park area on amount of within-neighbourhood walking [outcome]. **Table S13.** Housing type as a moderator of direct effects of neighbourhood residential density, mediators 1 and park area on frequency of outside-neighbourhood walking [outcome]. **Table S14.** Housing type as a moderator of direct effects of neighbourhood residential density, mediators 1 and park area on amount of outside-neighbourhood walking [outcome]. **Figure S1.** Directed acyclic graph (DAG) depicting the hypothesised relations between neighbourhood residential density, other environmental attributes, household car ownership, covariates and measures of transportation walking. Through the DAG, we identified which covariates to include in the statistical analyses to sufficiently control for potential confounders. **Figure S2.** Directed acyclic graph (DAG) depicting the hypothesised relations between neighbourhood residential density, other environmental attributes, household car ownership, covariates and measures of recreation walking. Through the DAG, we identified which covariates to include in the statistical analyses to sufficiently control for potential confounders.

## Data Availability

The data used in this paper include personal residential information as well as health-related information. Therefore, the data are not publicly available due to privacy concerns. Data are, however, available from the corresponding author upon reasonable request after obtaining permission from the Department of Health, Hong Kong SAR in accordance with the ethics policy statements related to the study protocol.

## Abbreviations

AIC: Akaike Information Criterion
ALECS: Active Lifestyle and the Environment in Chinese Seniors
e^*b*^: Exponentiated regression coefficient
CI: Confidence interval
EHCs: Elderly Health Centres
ESRI: Environmental System Research Institute
GAMMs: Generalised additive mixed models
GIS: Geographic Information Systems
GPS: Global Positioning System
HKSAR: Hong Kong Special Administrative Region
ICC: Intraclass correlation
NWQ-CS: Neighbourhood Walking Questionnaire for Chinese Seniors
SES: Socio-economic status
TPU: Tertiary Planning Unit
VIF: Variance inflation factor
