# Supplementary material for: How urban densification shapes walking behaviours in older community dwellers: a cross-sectional analysis of potential pathways of influence
Source: Int J Health Geogr. 2020 Apr 16;19:14. doi: 10.1186/s12942-020-00210-8 (PMC7164360; doi:10.1186/s12942-020-00210-8)
Supplement: Supplementary file 1 — Additional file 1: Table S1. Definitions of environmental variables (exposures), data sources and spatial resolution. Table S1. Outline of regression analyses. Table S2. Models 1M: Direct effect of neighbourhood residential density [exposure] on street intersection density, public transport density and density of four destination types [mediators 1]. Table S3. Model 2aM: Direct effects of neighbourhood residential density and mediators 1 on park area [mediator 2]. Table S4. Model 2bM: Direct effects of neighbourhood residential density and mediators 1 on household car ownership [mediator 2]. Table S5. Model 3M: Direct effects of neighbourhood residential density, mediators 1, park area and household car ownership [mediators 2] on frequency of within-neighbourhood walking [outcome]. Table S6. Model 4M: Direct effects of neighbourhood residential density, mediators 1, mediators 2 and domain-matching frequency of within-neighbourhood walking [mediator 3] on amount of within-neighbourhood walking [outcome]. Table S7. Model 5M: Direct effects of neighbourhood residential density, mediators 1, mediators 2, and mediator 3 on frequency of outside-neighbourhood walking [outcome]. Table S8. Model 6M: Direct effects of neighbourhood residential density, mediators 1, mediators 2, mediator 3, domain-matching frequency of outside-neighbourhood walking and amount of within-neighbourhood walking [mediators 4] on amount of outside-neighbourhood walking [outcome]. Table S9. Housing type as a moderator of total effects of neighbourhood residential density on walking measures. Table S10. Housing type as a moderator of direct effects of neighbourhood residential density and mediators 1 on household car ownership [mediator 2]. Table S11. Housing type as a moderator of direct effects of neighbourhood residential density, mediators 1 and park area on frequency of within-neighbourhood walking [outcome]. Table S12. Housing type as a moderator of direct effects of neighbourhood residential de [file 12942_2020_210_MOESM1_ESM.docx]

**Table S1. Definitions of environmental variables (exposures), data sources and spatial resolution**

| Environmental variable / feature | Definition | Data source | Spatial resolution |
| --- | --- | --- | --- |
| Participant street-network residential buffer | An irregularly shaped polygon around a participant’s home address (geocoded) that approximates neighbourhood boundaries. Buffer polygons were created for 800-m by tracing through unique street networks in all directions. We calculated the total land area (km^2^) of each participant residential buffer. All variables listed below were computed for each participant’s 800m street-network buffers. | Centreline road network data (RG1000)  Source: Lands Department | Vector data |
| Gross neighbouhood residential density | Number of residential households divided by the area of the participant street-network residential buffers, expressed as households per km^2^. | Number of households and area size  Source: Census and Statistics Department | Tertiary planning unit |
| Street intersection density | Number of three-and-more-way intersections divided by the area of the participant street-network residential buffers, expressed as intersections per km^2^. | Centreline road network data (RG1000)  Source: Lands Department | Line and point data |
| Civic and institutional destination density | Number of civic and institutional locations (e.g., government office, public services, school) divided by the area of the participant street-network residential buffers, expressed as number of destinations per km^2^. | Number of destinations (B10000)  Source: Lands Department | Point data |
| Entertainment density | Number of entertainment destinations (e.g., theatre, museum, community centre, art gallery) divided by the area of the participant street-network residential buffers, expressed as number of destinations per km^2^. | Number of destinations (B10000)  Source: Lands Department | Point data |
| Recreation density | Number of recreational destinations (e.g., sports centre, swimming pool, fitness club) divided by the area of the participant street-network residential buffers, expressed as number of destinations per km^2^. | Number of destinations (B10000)  Source: Lands Department | Point data |
| Food and retail density | Number of food and retail destinations (e.g., convenience store, cooked food stall, floating restaurants, market, supermarket, malls/shopping centrs, commercial complex) divided by the area of the participant street-network residential buffers, expressed as number of destinations per km^2^. | Number of destinations (B10000)  Source: Lands Department | Point data |
| Public transport density | Number of public transport points (e.g., bus terminals, ferry pier, ferry terminal, light rail station, green minibus terminus, railway station access) divided by the area of the participant street-network residential buffers, expressed as number of destinations per km^2^. | Number of destinations (B10000)  Source: Land and Transportation Departments | Point data |
| Park area | Total area (hectare) of public parks included the participant street-network residential buffers. | Park filled polygon data  (B5000)  Source: Lands Department | Polygon data |

**Detailed description of analytical steps**

*Total effects of urban densification on walking*

The total effects of urban densification (neighbourhood residential density) on each of the eight walking outcomes (e.g., weekly frequency of within-neighbourhood transportation walking) were first estimated (Step 1 in Table S1) using generalized additive mixed models (GAMMs). These GAMMs were adjusted for potential confounders, i.e., factors potentially associated with neighbourhood self-selection (choosing to live in low- or high-density areas) and walking outcomes. Potential confounders included age (Cerin et al., 2014; Schirmer et al., 2014), sex (Lewis and Baldassare, 2010; Zang et al., 2019), educational attainment (Cerin, Sit, et al., 2013; Lewis and Baldassare, 2010), living arrangements (Chad et al., 2005; Schirmer et al., 2014; Zhang, Barnett, Sit, et al., 2019), number of diagnosed chronic conditions (Barnett et al., 2016; James et al., 2015) and type of housing (Lewis and Baldassare, 2010; Zang et al., 2019). Type of housing (public, private and rental housing) was also considered as a moderator of densification-walking associations. As the allocation of public housing in Hong Kong is not based on individual preferences for residential location, neighbourhood self-selection is minimised among Hong Kong older adults living in public housing (Zang et al., 2019). Therefore, a comparison of associations among study participants residing in public, private and rental housing may help estimate the magnitude of neighbourhood self-selection bias among those in private and rental accommodation. Finally, although the stratified sampling strategy adopted in this study controlled for the potential confounding effects of TPU-level SES, TPU-level SES was included as a covariate to increase statistical power by accounting for unexplained outcome variance (Lingsma et al., 2010).

**Mediated and direct effects of urban densification on walking**

The presence of mediation effects was examined using the joint-significance test (MacKinnon and Luecken, 2008) and following the steps outlined in Table S1. According to this test, mediation is confirmed if the associations (regression coefficients) between an exposure and its mediator(s), and the exposure-adjusted associations between the mediator(s) and the outcome are statistically significant. This first entailed regressing street intersection density, public transport density and each of the destination density variables (mediators 1) onto residential density (Table S1). In step 2, we examined whether mediators 1 mediated the associations of residential density with park area and household car ownership (mediators 2). In step 3, the roles of mediators 1 and 2 in the associations between residential density and frequency of within-neighbourhood walking were examined. In steps 4 and 5, we estimated the mediating effects of mediators 1 and 2, and frequency of within-neighbourhood walking (mediator 3) on the associations of residential density with amount of within-neighbourhood and frequency of outside-neighbourhood walking. Finally, in step 6, amount of outside-neighbourhood walking was regressed on its domain-matching frequency of outside-neighbourhood walking, domain-matching amount of within-neighbourhood walking (mediators 4), mediators 1 and 2 and potential confounders. To estimate the potential impact of neighbourhood self-selection on the mediating effects, housing type was examined as a moderator of the above associations with household car ownership and walking outcomes. Significant moderation effects were probed by estimating associations at the three values of housing type (public, private and rental). All analyses were conducted in R version 3.4.3 (R Core Team, 2017) using the packages ‘mgcv’ version 1.8.22 (Wood, 2006) and ‘multcomp’ version 1.4.8 (Hothorn et al., 2008).

**References**

1. Barnett A, Cerin E, Zhang CJP, Sit CHP, Johnston JM, Cheung MMC, et al. Associations between the neighbourhood environment characteristics and physical activity in older adults with specific types of chronic conditions: the ALECS cross-sectional study. Int J Behav Nutr Phys Act. 2016;13:53. doi: 10.1186/s12966-016-0377-7.
2. Cerin E, Sit CH, Barnett A, Cheung MC, Chan WM. Walking for recreation and perceptions of the neighborhood environment in older Chinese urban dwellers. J Urban Health. 2013;90:56-66. doi: 10.1007/s11524-012-9704-8.
3. Cerin E, Sit CH, Barnett A, Johnston JM, Cheung MC, Chan WM. Ageing in an ultra-dense metropolis: perceived neighbourhood characteristics and utilitarian walking in Hong Kong elders. Public Health Nutr. 2014;17:225-232. doi: 10.1017/S1368980012003862.
4. Chad KE, Reeder BA, Harrison EL, Ashworth NL, Sheppard SM, Schultz SL, et al. Profile of physical activity levels in community-dwelling older adults. Med Sci Sports Exerc. 2005;37:1774-1784. doi: 10.1249/01.mss.0000181303.51937.9c.
5. Hothorn T, Bretz F, Westfall P. Simultaneous inference in general parametric models. Biometrical J. 2008;50:346—363. doi: 10.1002/bimj.200810425.
6. James P, Hart JE, Arcaya MC, Feskanich D, Laden F, Subramanian SV. Neighborhood self-selection: the role of pre-move health factors on the built and socioeconomic environment. Int J Environ Res Public Health. 2015;12:12489-12504. doi: 10.3390/ijerph121012489.
7. Lewis PG, Baldassare M. The complexity of public attitudes toward compact development. J Am Planning Assoc. 2010;76:219-237. doi: 10.1080/01944361003646471.
8. Lingsma H, Roozenbeek B, Steyerberg E, IMPACT investigators. Covariate adjustment increases statistical power in randomized controlled trials. J Clin Epidemiol. 2010;63:1391. doi: 10.1016/j.jclinepi.2010.05.003.
9. MacKinnon DP, Luecken LJ. How and for whom? Mediation and moderation in health psychology. Health Psychol. 2008;27(2S):S99-S100. doi: 10.1037/0278-6133.27.2(Suppl.) S99.
10. R Core Team. R: A language and environment for statistical computing. Vienna, Austria: R Foundation for Statistical Computing. 2017. https://www.R-project.org/.
11. Schirmer PM, van Eggermond MAB, Axhausen KW. The role of location in residential location choice models: a review of literature. J Transport Land Use. 2014;7:3-21. doi: 10.5198/jtlu.v7i2.740.
12. Wood SN. Generalised Additive Models: An Introduction with R, 2nd ed. Boca Raton, FL: Chapman & Hall/CRC. 2006.
13. Zang P, Lu Y, Ma J, Xie B, Wang R, Liu Y. Disentangling residential self-selection from impacts of built environment characteristics on travel behaviors for older adults. Soc Sci Med. 2019;238:112515. doi: 10.1016/j.socscimed.2019.112515.
14. Zhang CJP, Barnett A, Sit CHP, Lai PC, Johnston JM, Lee RSY, et al. To what extent does physical activity explain the associations between neighborhood environment and depressive symptoms in older adults living in an Asian metropolis? Mental Health Phys Act. 2019;16: 96-104. doi: 10.1016/j.mhpa.2018.11.005.

**Table S1. Outline of regression analyses**

| Step | Effect estimated | Covariates | Regression models |
| --- | --- | --- | --- |
| *Estimation of total effects of densification on walking* | |  |  |
| 1T* | Total effects of neighbourhood residential density on eight walking measures | Age, chronic health conditions, educational attainment, sex, TPU-level SES and housing type | Eight separate GAMMs, one for each walking outcome. GAMMs with negative binomial variance and logarithmic link functions for walking frequency. GAMMs with Gamma variance and logarithmic link function for amount of walking. |
| *Estimation of direct and mediated effects of densification on walking* | |  |  |
| 1M | Direct effect of neighbourhood residential density [exposure] on street intersection density, public transport density and density of four destination types [mediators 1] | None | Six separate GAMMs, one for each environmental attribute (i.e., mediator 1). GAMMs with Gaussian or Gamma variance and logarithmic link functions. |
| 2aM | Direct effects of neighbourhood residential density and mediators 1 on park area [mediator 2]. | None | A single GAMM with Gamma variance and logarithmic link functions. |
| 2bM* | Direct effects of neighbourhood residential density and mediators 1 on household car ownership [mediator 2] | Age, educational attainment, sex, TPU-level SES, living arrangements, housing type | A single GAMM with binomial variance and logarithmic link functions.^a^ |
| 3M* | Direct effects of neighbourhood residential density, mediators 1, park area and household car ownership [mediators 2] on frequency of within-neighbourhood walking [outcome] | Age, chronic health conditions, educational attainment, living arrangements, TPU-level SES and housing type | Two GAMMs, one per walking domain, with negative binomial variance and logarithmic link functions.^a^ |
| 4M* | Direct effects of neighbourhood residential density, mediators 1, mediators 2 and domain-matching frequency of within-neighbourhood walking [mediator 3] on amount of within-neighbourhood walking [outcome] | Age, chronic health conditions, educational attainment, living arrangements, TPU-level SES and housing type | Two GAMMs, one per walking domain, with Gamma variance and logarithmic link functions.^a^ |
| 5M* | Direct effects of neighbourhood residential density, mediators 1, mediators 2, and mediator 3 on frequency of outside-neighbourhood walking [outcome] | Age, chronic health conditions, educational attainment, sex, TPU-level SES and housing type | Two GAMMs, one per walking domain, with negative binomial variance and logarithmic link functions.^a^ |
| 6M* | Direct effects of neighbourhood residential density, mediators 1, mediators 2, mediator 3, domain-matching frequency of outside-neighbourhood walking and amount of within-neighbourhood walking [mediators 4] on amount of outside-neighbourhood walking [outcome] | Age, chronic health conditions, educational attainment, living arrangements, sex, TPU-level SES and housing type | Two GAMMs, one per walking domain, with Gamma variance and logarithmic link functions.^a^ |

*Note.* TPU, Tertiary Planning Unit; SES, socio-economic status; GAMM, generalised additive mixed model.

* The moderating effects of housing type on exposure-outcome or mediator-outcome associations were examined by adding two-way interaction terms to these models.

^a^ To assess the impact of collinearity on the findings from the multiple-environmental-variable models, single environmental variable models were also estimated.

**Table S2. Models 1M: Direct effect of neighbourhood residential density [exposure] on street intersection density, public transport density and density of four destination types [mediators 1]**

| Mediators 1 [response variables] | *F*-ratio (degrees if freedom) | *p-*value |
| --- | --- | --- |
| Street intersection density (destinations / km^2^) | *F* (3.49, 904.51) = 28.70; see Figure 3 – panel C | <.001 |
| Public transport density (destinations / km^2^) | *F* (3.85, 904.15) = 44.09; see Figure 3 – panel B | <.001 |
| Entertainment density (destinations / km^2^) | *F* (4.59, 903.41) = 29.55; see Figure 3 – panel A | <.001 |
| Recreation density (destinations / km^2^) | *F* (6.82, 901.18) = 34.45; see Figure 3 – panel D | <.001 |
| Food and retail density (destinations / km^2^) | *F* (2.91, 905.09) = 77.76; see Figure 3 – panel E | <.001 |
| Civic and institutional destination density (destinations / km^2^) | *F* (3.71, 904.29) = 78.70; see Figure 3 – panel F | <.001 |

Note: *F,* *F*-ratio of smooth term (curvilinear relationship). Generalised additive mix models with Gamma or Gaussian variance and logarithmic link functions. Random intercepts at the Tertiary Planning Unit level.

**Table S3. Model 2aM: Direct effects of neighbourhood residential density and mediators 1 on park area [mediator 2]**

| Predictors of park area (ha) | e*^b^* (95% CI) or *F*-ratio (df) | *p-*value |
| --- | --- | --- |
| Neighbourhood residential density (‘000 dwellings/km^2^) | *F* (3.59, 896.77) = 14.57; see Figure 4 – panel A | <.001 |
| Street intersection density (destinations / km^2^) | 1.002 (1.001, 1.004) | .008 |
| Public transport density (destinations / km^2^) | *F* (2.65, 896.77) = 7.80; see Figure 4 – panel B | .001 |
| Entertainment density (destinations / km^2^) | 0.983 (0.973, 0.993) | .001 |
| Recreation density (destinations / km^2^) | 0.999 (0.996, 1.002) | .395 |
| Food and retail density (destinations / km^2^) | 0.999 (0.997, 1.001) | .411 |
| Civic and institutional destination density (destinations / km^2^) | 1.001 (0.999, 1.003) | .389 |

Note: ha, hectare; e*^b^*, exponentiated regression coefficient; CI, confidence intervals; *F,* *F*-ratio of smooth term (curvilinear relationship); df = degrees of freedom. Generalised additive mix model with Gamma variance and logarithmic link functions. Random intercepts at the Tertiary Planning Unit level.

**Table S4. Model 2bM: Direct effects of neighbourhood residential density and mediators 1 on household car ownership [mediator 2]**

| Predictors of household car ownership (ref: no car) | e*^b^* (95% CI) | *p-*value |
| --- | --- | --- |
| Neighbourhood residential density (‘000 dwellings/km^2^) | 0.993 (0.973, 1.013) | .461 |
| Street intersection density (destinations / km^2^) | 0.996 (0.992, 0.999) | .041 |
| Public transport density (destinations / km^2^) | 0.997 (0.980, 1.015) | .771 |
| Entertainment density (destinations / km^2^) | 0.993 (0.964, 1.022) | .636 |
| Recreation density (destinations / km^2^) | 0.996 (0.988, 1.004) | .367 |
| Food and retail density (destinations / km^2^) | 0.997 (0.994, 0.999) | .029 |
| Civic and institutional destination density (destinations / km^2^) | 0.995 (0.990, 1.000) | .079 |
| Park area (ha) | 0.975 (0.980, 1.060) | .550 |

Note: ref, reference category; e*^b^*, exponentiated regression coefficient; CI, confidence intervals. Generalised additive mix model with binomial variance and logarithmic link functions. Random intercepts at the Tertiary Planning Unit (TPU) level. Model adjusted for age, educational attainment, sex, TPU-level SES, living arrangements, housing type.

**Table S5. Model 3M: Direct effects of neighbourhood residential density, mediators 1, park area and household car ownership [mediators 2] on frequency of within-neighbourhood walking [outcome]**

| Predictors | e*^b^* (95% CI) | *p-*value |
| --- | --- | --- |
| *Model of transportation walking* |  |  |
| Neighbourhood residential density (‘000 dwellings/km^2^) | 1.001 (0.992, 1.009) | .906 |
| Street intersection density (destinations / km^2^) | 1.000 (0.998, 1.001) | .687 |
| Public transport density (destinations / km^2^) | 1.004 (0.996, 1.013) | .313 |
| Entertainment density (destinations / km^2^) | 0.987 (0.974, 1.001) | .072 |
| Recreation density (destinations / km^2^) | 1.000 (0.997, 1.004) | .820 |
| Food and retail density (destinations / km^2^) | 1.004 (1.002, 1.007) | .002 |
| Civic and institutional destination density (destinations / km^2^) | 0.999 (0.996, 1.002) | .453 |
| Park area (ha) | 0.992 (0.960, 1.026) | .660 |
| Household car ownership (ref: no car) | 0.864 (0.766, 0.976) | .019 |
| *Model of recreation walking* |  |  |
| Neighbourhood residential density (‘000 dwellings/km^2^) | 1.000 (0.987, 1.014) | .957 |
| Street intersection density (destinations / km^2^) | 0.997 (0.995, 0.999) | .049 |
| Public transport density (destinations / km^2^) | 0.987 (0.976, 0.998) | .017 |
| Entertainment density (destinations / km^2^) | 0.992 (0.973, 1.012) | .452 |
| Recreation density (destinations / km^2^) | 1.006 (1.001, 1.011) | .025 |
| Food and retail density (destinations / km^2^) | 1.003 (1.000, 1.006) | .041 |
| Civic and institutional destination density (destinations / km^2^) | 1.001 (0.997, 1.005) | .689 |
| Park area (ha) | 1.031 (0.989, 1.076) | .155 |
| Household car ownership (ref: no car) | 0.947 (0.816, 1.098) | .470 |

Note: e*^b^*, exponentiated regression coefficient; CI, confidence intervals. Generalised additive mix models with negative binomial variance and logarithmic link functions. Random intercepts at the Tertiary Planning Unit (TPU) level. Model adjusted for age, chronic health conditions, educational attainment, living arrangements, TPU-level SES and housing type.

**Table S6. Model 4M: Direct effects of neighbourhood residential density, mediators 1, mediators 2 and domain-matching frequency of within-neighbourhood walking [mediator 3] on amount of within-neighbourhood walking [outcome]**

| Predictors | e*^b^* (95% CI) or *F*-ratio (df) | *p* value |
| --- | --- | --- |
| *Model of transportation walking* |  |  |
| Neighbourhood residential density (‘000 dwellings/km^2^) | 1.018 (1.003, 1.033) | .021 |
| Street intersection density (destinations / km^2^) | 0.999 (0.996, 1.002) | .529 |
| Public transport density (destinations / km^2^) | 0.985 (0.971, 0.998) | .029 |
| Entertainment density (destinations / km^2^) | 1.004 (0.982, 1.028) | .703 |
| Recreation density (destinations / km^2^) | 0.992 (0.985, 0.998) | .007 |
| Food and retail density (destinations / km^2^) | *F* (4.44, 884.72) = 3.21; see Figure 5 – panel A | .010 |
| Civic and institutional destination density (destinations / km^2^) | 0.997 (0.992, 1.001) | .133 |
| Park area (ha) | 0.973 (0.924, 1.024) | .286 |
| Household car ownership (ref: no car) | 0.822 (0.686, 0.986) | .034 |
| Frequency of within-neighbourhood transportation walking (times/week) | *F* (1.99, 884.72) = 304.20; see Figure 5 – panel B | <.001 |
| *Model of recreation walking* |  |  |
| Neighbourhood residential density (‘000 dwellings/km^2^) | 1.003 (0.984, 1.023) | .755 |
| Street intersection density (destinations / km^2^) | 0.998 (0.994, 1.001) | .218 |
| Public transport density (destinations / km^2^) | 1.014 (0.996, 1.034) | .133 |
| Entertainment density (destinations / km^2^) | 1.018 (0.988, 1.050) | .247 |
| Recreation density (destinations / km^2^) | 1.002 (0.993, 1.011) | .661 |
| Food and retail density (destinations / km^2^) | 1.002 (0.996, 1.008) | .549 |
| Civic and institutional destination density (destinations / km^2^) | 0.996 (0.990, 1.002) | .191 |
| Park area (ha) | 0.997 (0.976, 1.018) | .776 |
| Household car ownership (ref: no car) | 0.992 (0.764, 1.290) | .954 |
| Frequency of within-neighbourhood recreation walking (times/week) | 2.027 (1.968, 2.088) | <.001 |

Note: e*^b^*, exponentiated regression coefficient; CI, confidence intervals; *F,* *F*-ratio of smooth term (curvilinear relationship); df, degrees of freedom. Generalised additive mix models with Gamma variance and logarithmic link functions. Random intercepts at the Tertiary Planning Unit (TPU) level. Model adjusted for age, chronic health conditions, educational attainment, living arrangements, TPU-level SES and housing type.

**Table S7. Model 5M: Direct effects of neighbourhood residential density, mediators 1, mediators 2, and mediator 3 on frequency of outside-neighbourhood walking [outcome]**

| Predictors | e*^b^* (95% CI) or *F*-ratio (df) | *p* value |
| --- | --- | --- |
| *Model of transportation walking* |  |  |
| Neighbourhood residential density (‘000 dwellings/km^2^) | 1.004 (0.989, 1.019) | .599 |
| Street intersection density (destinations / km^2^) | 1.004 (1.001, 1.007) | .010 |
| Public transport density (destinations / km^2^) | 1.004 (0.990, 1.018) | .570 |
| Entertainment density (destinations / km^2^) | 1.007 (0.984, 1.030) | .554 |
| Recreation density (destinations / km^2^) | 0.999 (0.993, 1.005) | .844 |
| Food and retail density (destinations / km^2^) | 0.996 (0.992, 1.001) | .093 |
| Civic and institutional destination density (destinations / km^2^) | 1.001 (0.996, 1.005) | .731 |
| Park area (ha) | 0.974 (0.926, 1.026) | .322 |
| Household car ownership (ref: no car) | 0.969 (0.808, 1.161) | .730 |
| Frequency of within-neighbourhood transportation walking (times/week) | *F* (3.72, 904.28) = 4.22; see Figure 6 | .002 |
| *Model of recreation walking* |  |  |
| Neighbourhood residential density (‘000 dwellings/km^2^) | 1.003 (0.992, 1.014) | .625 |
| Street intersection density (destinations / km^2^) | 1.002 (1.000, 1.004) | .048 |
| Public transport density (destinations / km^2^) | 1.002 (0.992, 1.013) | .700 |
| Entertainment density (destinations / km^2^) | 1.000 (0.983, 1.018) | .996 |
| Recreation density (destinations / km^2^) | 0.999 (0.994, 1.004) | .681 |
| Food and retail density (destinations / km^2^) | 1.001 (0.998, 1.004) | .510 |
| Civic and institutional destination density (destinations / km^2^) | 1.001 (0.998, 1.005) | .469 |
| Park area (ha) | 0.999 (0.960, 1.040) | .976 |
| Household car ownership (ref: no car) | 0.997 (0.867, 1.153) | .996 |
| Frequency of within-neighbourhood recreation walking (times/week) | 0.976 (0.960, 0.992) | .003 |

Note: e*^b^*, exponentiated regression coefficient; CI, confidence intervals; *F,* *F*-ratio of smooth term (curvilinear relationship); df = degrees of freedom. Generalised additive mix models with negative binomial variance and logarithmic link functions. Random intercepts at the Tertiary Planning Unit level. Model adjusted for age, chronic health conditions, educational attainment, sex, TPU-level SES and housing type.

**Table S8. Model 6M: Direct effects of neighbourhood residential density, mediators 1, mediators 2, mediator 3, domain-matching frequency of outside-neighbourhood walking and amount of within-neighbourhood walking [mediators 4] on amount of outside-neighbourhood walking [outcome]**

| Predictors | e*^b^* (95% CI) | *p* value |
| --- | --- | --- |
| *Model of transportation walking* |  |  |
| Neighbourhood residential density (‘000 dwellings/km^2^) | 0.995 (0.973, 1.017) | .668 |
| Street intersection density (destinations / km^2^) | 1.002 (0.997, 1.006) | .466 |
| Public transport density (destinations / km^2^) | 0.999 (0.978, 1.020) | .900 |
| Entertainment density (destinations / km^2^) | 0.999 (0.965, 1.034) | .952 |
| Recreation density (destinations / km^2^) | 1.005 (0.996, 1.014) | .298 |
| Food and retail density (destinations / km^2^) | 0.997 (0.932, 1.067) | .418 |
| Civic and institutional destination density (destinations / km^2^) | 0.996 (0.989, 1.002) | .206 |
| Park area (ha) | 1.007 (0.929, 1.092) | .689 |
| Household car ownership (ref: no car) | 1.225 (0.914, 1.640) | .174 |
| Frequency of within-neighbourhood transportation walking (times/week) | 0.984 (0.965, 1.003) | .100 |
| Amount of within-neighbourhood transportation walking (min/week) | 1.001 (1.001, 1.002) | <.001 |
| Frequency of outside-neighbourhood transportation walking (times/week) | 1.631 (1.585, 1.679) | <.001 |
| *Model of recreation walking* |  |  |
| Neighbourhood residential density (‘000 dwellings/km^2^) | 1.006 (0.975, 1.037) | .717 |
| Street intersection density (destinations / km^2^) | 0.999 (0.993, 1.005) | .834 |
| Public transport density (destinations / km^2^) | 1.042 (1.011, 1.074) | .007 |
| Entertainment density (destinations / km^2^) | 1.009 (0.961, 1.060) | .721 |
| Recreation density (destinations / km^2^) | 0.999 (0.986, 1.013) | .885 |
| Food and retail density (destinations / km^2^) | 0.994 (0.985, 1.003) | .239 |
| Civic and institutional destination density (destinations / km^2^) | 1.003 (0.993, 1.013) | .602 |
| Park area (ha) | 0.941 (0.835, 1.060) | .315 |
| Household car ownership (ref: no car) | 0.644 (0.420, 0.988) | .044 |
| Frequency of within-neighbourhood recreation walking (times/week) | 1.014 (0.943, 1.091) | .701 |
| Amount of within-neighbourhood recreation walking (min/week) | 3.992 (3.592, 4.436) | <.001 |
| Frequency of outside-neighbourhood recreation walking (times/week) | 1.000 (0.999, 1.002) | .520 |

Note: e*^b^*, exponentiated regression coefficient; CI, confidence intervals. Generalised additive mix models with Gamma variance and logarithmic link functions. Random intercepts at the Tertiary Planning Unit level. Model adjusted for age, chronic health conditions, educational attainment, living arrangements, sex, TPU-level SES and housing type.

**Table S9. Housing type as a moderator of total effects of neighbourhood residential density on walking measures**

|  | Transportation walking | | Recreation walking | |
| --- | --- | --- | --- | --- |
| Walking measure | *F* (2, 898) | *p* | *F* (2, 898) | *p* |
| Frequency (times/week) – within neighbourhood | 2.00 | .137 | 0.44 | .643 |
| Amount (min/week) – within neighbourhood | 1.96 | .142 | 1.37 | .254 |
| Frequency (times/week) – outside neighbourhood | 0.60 | .577 | 0.44 | .644 |
| Amount (min/week) – outside neighbourhood | 0.62 | .536 | 1.78 | .169 |

Note: *F, F*-ratio interaction terms of residential density by private housing and residential density by rental housing (reference category: public housing); *p,* *p*-value.

**Table S10.** **Housing type as a moderator of direct effects of neighbourhood residential density and mediators 1 on household car ownership [mediator 2]**

| Environmental attributes | *F* (2, 891) | *p* value |
| --- | --- | --- |
| Neighbourhood residential density (‘000 dwellings/km^2^) | 2.70 | .068 |
| Street intersection density (destinations / km^2^) | 0.69 | .504 |
| Public transport density (destinations / km^2^) | 2.25 | .106 |
| Entertainment density (destinations / km^2^) | 0.01 | .998 |
| Recreation density (destinations / km^2^) | 0.40 | .669 |
| Food and retail density (destinations / km^2^) | 1.28 | .278 |
| Civic and institutional destination density (destinations / km^2^) | 0.14 | .869 |
| Park area (ha) | 0.01 | .998 |

Note: *F, F*-ratio interaction terms of environmental attributes by private housing and environmental attributes by rental housing (reference category: public housing); *p,* *p*-value.

**Table S11.Housing type as a moderator of direct effects of neighbourhood residential density, mediators 1 and park area on frequency of within-neighbourhood walking [outcome]**

| Environmental attributes | *F* (2, 889) | *p* value |
| --- | --- | --- |
| *Model of transportation walking* |  |  |
| Neighbourhood residential density (‘000 dwellings/km^2^) | 2.31 | .100 |
| Street intersection density (destinations / km^2^) | 2.72 | .066 |
| Public transport density (destinations / km^2^) | 0.60 | .547 |
| Entertainment density (destinations / km^2^) | 0.22 | .800 |
| Recreation density (destinations / km^2^) | 2.27 | .104 |
| Food and retail density (destinations / km^2^) | 1.16 | .313 |
| Civic and institutional destination density (destinations / km^2^) | 2.09 | .124 |
| Park area (ha) | 1.37 | .254 |
| *Model of recreation walking* |  |  |
| Neighbourhood residential density (‘000 dwellings/km^2^) | 0.24 | .789 |
| Street intersection density (destinations / km^2^) | 0.05 | .951 |
| Public transport density (destinations / km^2^) | 0.30 | .745 |
| Entertainment density (destinations / km^2^) | 0.18 | .839 |
| Recreation density (destinations / km^2^) | 0.73 | .485 |
| Food and retail density (destinations / km^2^) | 0.62 | .537 |
| Civic and institutional destination density (destinations / km^2^) | 0.03 | .974 |
| Park area (ha) | 2.55 | .079 |

Note: *F, F*-ratio interaction terms of environmental attributes by private housing and environmental attributes by rental housing (reference category: public housing); *p,* *p*-value.

**Table S12. Housing type as a moderator of direct effects of neighbourhood residential density, mediators 1 and park area on amount of within-neighbourhood walking [outcome]**

| Environmental attributes | *F* (2, 887) | *p* value |
| --- | --- | --- |
| *Model of transportation walking* |  |  |
| Neighbourhood residential density (‘000 dwellings/km^2^) | 2.83 | .060 |
| Street intersection density (destinations / km^2^) | 2.79 | .062 |
| Public transport density (destinations / km^2^) | 1.51 | .220 |
| Entertainment density (destinations / km^2^) | 0.35 | .703 |
| Recreation density (destinations / km^2^) | 2.72 | .067 |
| Food and retail density (destinations / km^2^) | 1.80 | .165 |
| Civic and institutional destination density (destinations / km^2^) | 2.48 | .084 |
| Park area (ha) | 1.38 | .252 |
| *Model of recreation walking* |  |  |
| Neighbourhood residential density (‘000 dwellings/km^2^) | 0.38 | .682 |
| Street intersection density (destinations / km^2^) | 0.20 | .821 |
| Public transport density (destinations / km^2^) | 0.34 | .713 |
| Entertainment density (destinations / km^2^) | 0.69 | .504 |
| Recreation density (destinations / km^2^) | 1.13 | .322 |
| Food and retail density (destinations / km^2^) | 0.67 | .512 |
| Civic and institutional destination density (destinations / km^2^) | 0.27 | .765 |
| Park area (ha) | 2.65 | .071 |

Note: *F, F*-ratio interaction terms of environmental attributes by private housing and environmental attributes by rental housing (reference category: public housing); *p,* *p*-value.

**Table S13. Housing type as a moderator of direct effects of neighbourhood residential density, mediators 1 and park area on frequency of outside-neighbourhood walking [outcome]**

| Environmental attributes | *F* (2, 886.3) | *p* value |
| --- | --- | --- |
| *Model of transportation walking* |  |  |
| Neighbourhood residential density (‘000 dwellings/km^2^) | 0.87 | .418 |
| Street intersection density (destinations / km^2^) | 2.07 | .128 |
| Public transport density (destinations / km^2^) | 1.11 | .329 |
| Entertainment density (destinations / km^2^) | 2.16 | .116 |
| Recreation density (destinations / km^2^) | 0.92 | .400 |
| Food and retail density (destinations / km^2^) | 0.41 | .661 |
| Civic and institutional destination density (destinations / km^2^) | 0.89 | .413 |
| Park area (ha) | 0.44 | .645 |
| *Model of recreation walking* | *F* (2,889) |  |
| Neighbourhood residential density (‘000 dwellings/km^2^) | 0.06 | .942 |
| Street intersection density (destinations / km^2^) | 0.29 | .750 |
| Public transport density (destinations / km^2^) | 0.26 | .772 |
| Entertainment density (destinations / km^2^) | 0.61 | .546 |
| Recreation density (destinations / km^2^) | 1.13 | .324 |
| Food and retail density (destinations / km^2^) | 0.27 | .767 |
| Civic and institutional destination density (destinations / km^2^) | 0.22 | .801 |
| Park area (ha) | 0.03 | .973 |

Note: *F, F*-ratio interaction terms of environmental attributes by private housing and environmental attributes by rental housing (reference category: public housing); *p,* *p*-value.

**Table S14. Housing type as a moderator of direct effects of neighbourhood residential density, mediators 1 and park area on amount of outside-neighbourhood walking [outcome]**

| Environmental attributes | *F* (2, 886) | *p* value |
| --- | --- | --- |
| *Model of transportation walking* |  |  |
| Neighbourhood residential density (‘000 dwellings/km^2^) | 0.92 | .397 |
| Street intersection density (destinations / km^2^) | 0.60 | .550 |
| Public transport density (destinations / km^2^) | 0.06 | .938 |
| Entertainment density (destinations / km^2^) | 0.71 | .493 |
| Recreation density (destinations / km^2^) | 0.24 | .790 |
| Food and retail density (destinations / km^2^) | 0.28 | .755 |
| Civic and institutional destination density (destinations / km^2^) | 1.25 | .287 |
| Park area (ha) | 0.06 | .946 |
| *Model of recreation walking* |  |  |
| Neighbourhood residential density (‘000 dwellings/km^2^) | 0.54 | .581 |
| Street intersection density (destinations / km^2^) | 0.70 | .498 |
| Public transport density (destinations / km^2^) | 1.85 | .158 |
| Entertainment density (destinations / km^2^) | 2.35 | .096 |
| Recreation density (destinations / km^2^) | 0.16 | .853 |
| Food and retail density (destinations / km^2^) | 1.70 | .184 |
| Civic and institutional destination density (destinations / km^2^) | 1.74 | .176 |
| Park area (ha) | 0.01 | .996 |

Note: *F, F*-ratio interaction terms of environmental attributes by private housing and environmental attributes by rental housing (reference category: public housing); *p,* *p*-value.


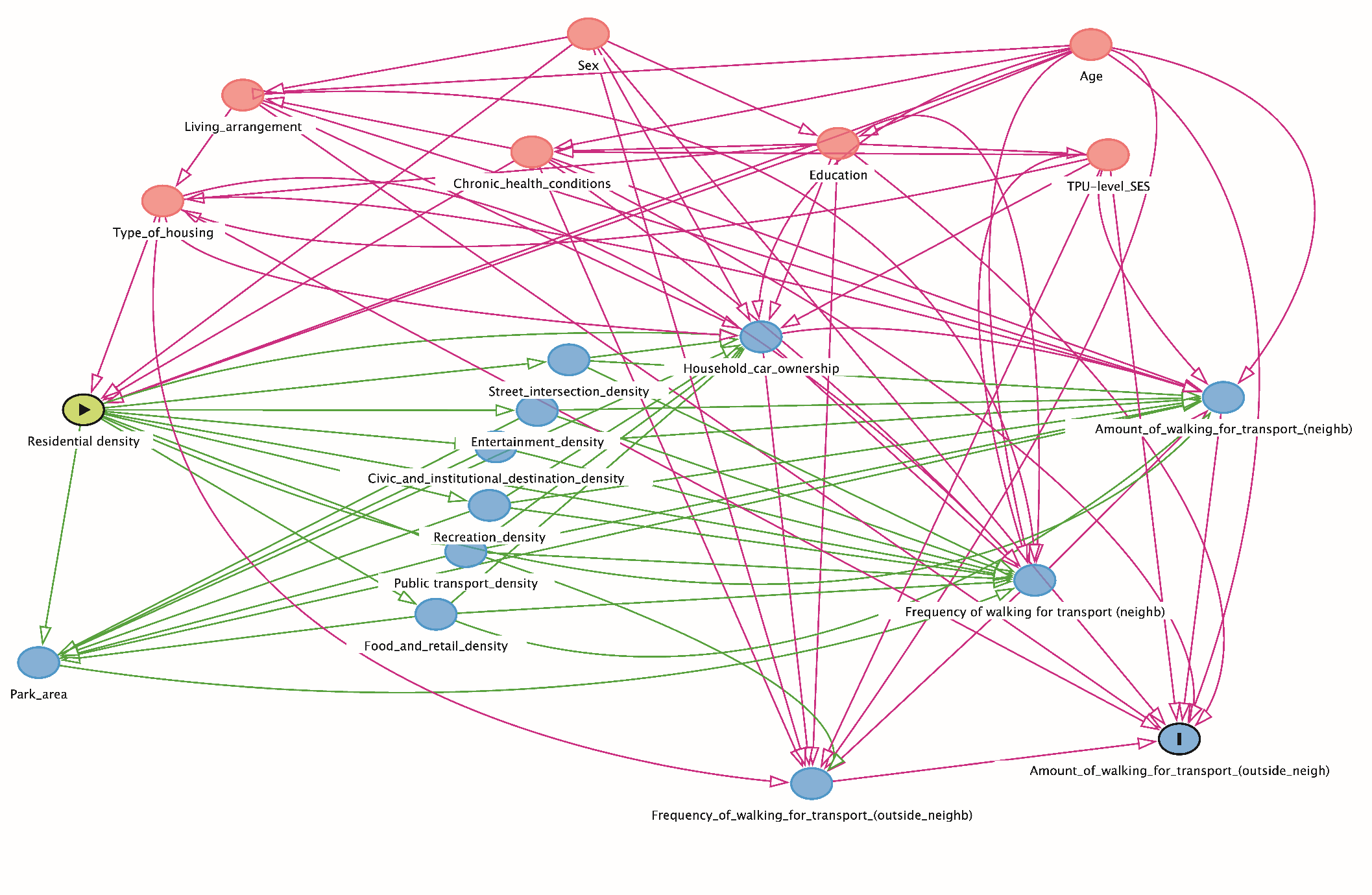


**Figure S1.** Directed acyclic graph (DAG) depicting the hypothesised relations between neighbourhood residential density, other environmental attributes, household car ownership, covariates and measures of transportation walking. Through the DAG, we identified which covariates to include in the statistical analyses to sufficiently control for potential confounders.


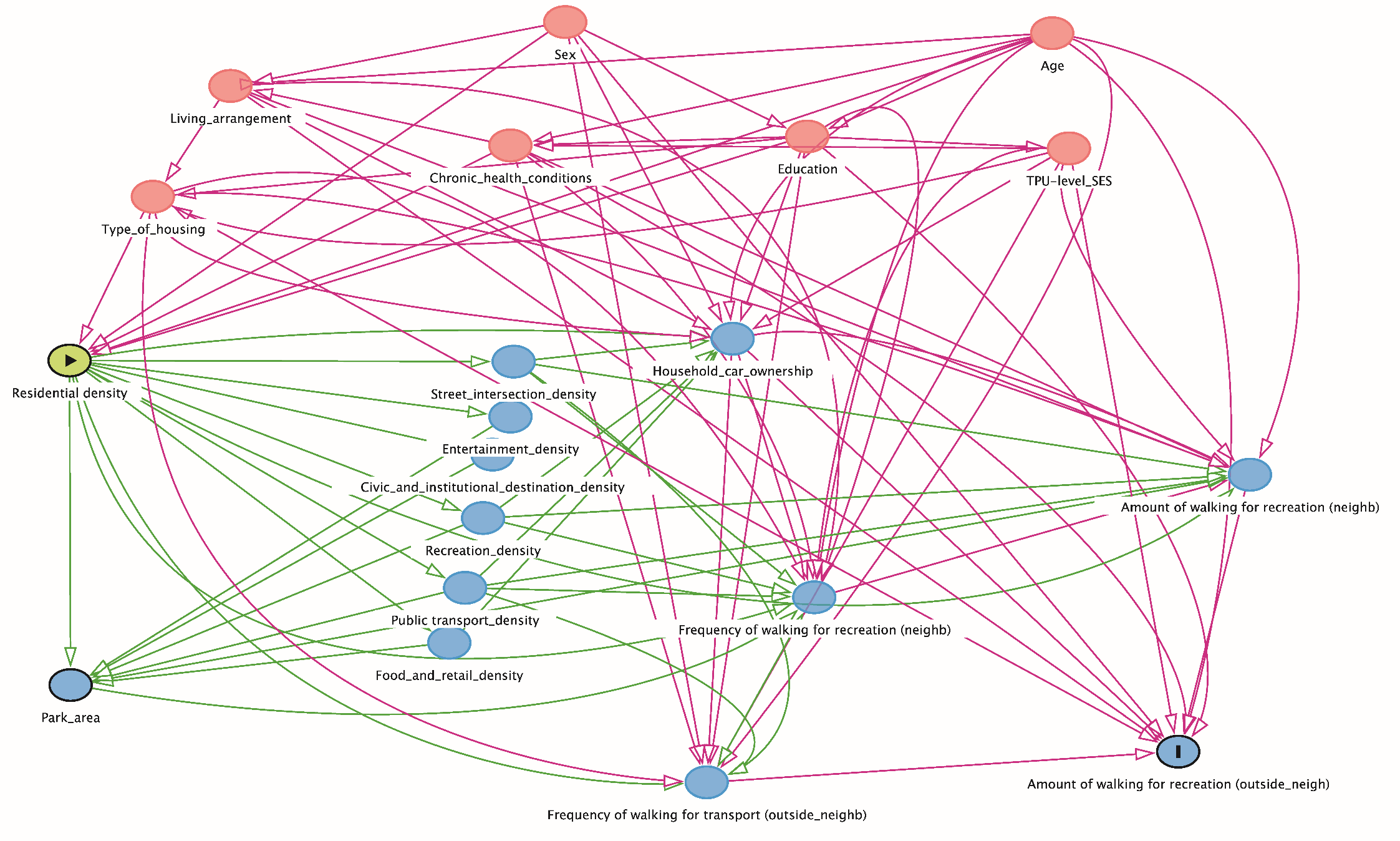


**Figure S2.** Directed acyclic graph (DAG) depicting the hypothesised relations between neighbourhood residential density, other environmental attributes, household car ownership, covariates and measures of recreation walking. Through the DAG, we identified which covariates to include in the statistical analyses to sufficiently control for potential confounders.
